# VISTA deficiency drives an activated NK cell phenotype with impaired cytotoxic function

**DOI:** 10.1093/jimmun/vkag282

**Published:** 2026-11-13

**Authors:** Junyan Cao, Shima Shahbaz, Ako Azimi, Amirhossein Rahmati, Emmanuel T Alimo, John Walker, Carrie Ye, Shokrollah Elahi

**Affiliations:** Mike Petryk School of Dentistry, Division of Foundational Sciences, University of Alberta, Edmonton, AB, Canada; Mike Petryk School of Dentistry, Division of Foundational Sciences, University of Alberta, Edmonton, AB, Canada; Mike Petryk School of Dentistry, Division of Foundational Sciences, University of Alberta, Edmonton, AB, Canada; Mike Petryk School of Dentistry, Division of Foundational Sciences, University of Alberta, Edmonton, AB, Canada; Mike Petryk School of Dentistry, Division of Foundational Sciences, University of Alberta, Edmonton, AB, Canada; Department of Oncology, University of Alberta, & Cross Cancer Institute, Edmonton, AB, Canada; Cancer Research Institute of Northern Alberta, University of Alberta, Edmonton, AB, Canada; Division of Rheumatology, Department of Medicine, University of Alberta, Edmonton, AB, Canada; Mike Petryk School of Dentistry, Division of Foundational Sciences, University of Alberta, Edmonton, AB, Canada; Cancer Research Institute of Northern Alberta, University of Alberta, Edmonton, AB, Canada; Li Ka Shing Institute of Virology, University of Alberta, Edmonton, AB, Canada; Women and Children Health Research Institute, University of Alberta, Edmonton, AB, Canada; Glycomics Institute of Alberta, University of Alberta, Edmonton, AB, Canada; Alberta Transplant Institute, Faculty of Medicine and Dentistry, University of Alberta, Edmonton, AB, Canada

**Keywords:** cancer, cytotoxicity, metabolism, NK cells, VISTA

## Abstract

Natural killer (NK) cell effector function requires coordinated receptor signaling, cytotoxic granule release, and metabolic fitness. V-domain Ig suppressor of T cell activation (VISTA) is an immune checkpoint implicated in immune regulation, but its role in NK cell biology remains incompletely defined. Using VISTA-knockout (VKO) and wild-type mice, murine EO771 mammary tumor-bearing mice, and peripheral blood NK cells from human healthy controls and non-small cell lung cancer patients, we examined how VISTA expression or deficiency shapes NK cell phenotype and function. VISTA was detected on a subset of murine NK cells with an activated phenotype. In contrast, systemic VISTA deficiency expanded splenic NK cell numbers but produced phenotypic activation with impaired degranulation and reduced cytotoxicity. Bulk RNA sequencing showed that VKO NK cells upregulated activation, cytokine, DNA damage-response, type I interferon, NF-κB, and inflammasome-associated programs, while repressing ER-to-Golgi/degranulation pathways, glycolysis, fatty acid metabolism, oxidative phosphorylation, and mitochondrial quality-control genes. Functionally, VKO NK cells showed reduced mitochondrial mass, increased mitochondrial reactive oxygen species, and altered membrane potential. In the tumor model, VKO mice exhibited reduced NK cell infiltration and CXCR3 expression within tumors, and tumor-infiltrating NK cells displayed impaired cytotoxicity. NK cell depletion did not alter tumor control in VKO mice, despite enhancing tumor growth in wild-type mice. In humans, VISTA expression marked activated NK cell subsets with higher IFN-γ, CD69, and CD107a and reduced intracellular granzyme B/perforin. Thus, systemic VISTA deficiency uncouples NK cell activation from metabolic and cytolytic competence, whereas VISTA expression identifies activated human NK cell subsets.

## Graphical abstract

**Graphical Abstract:**
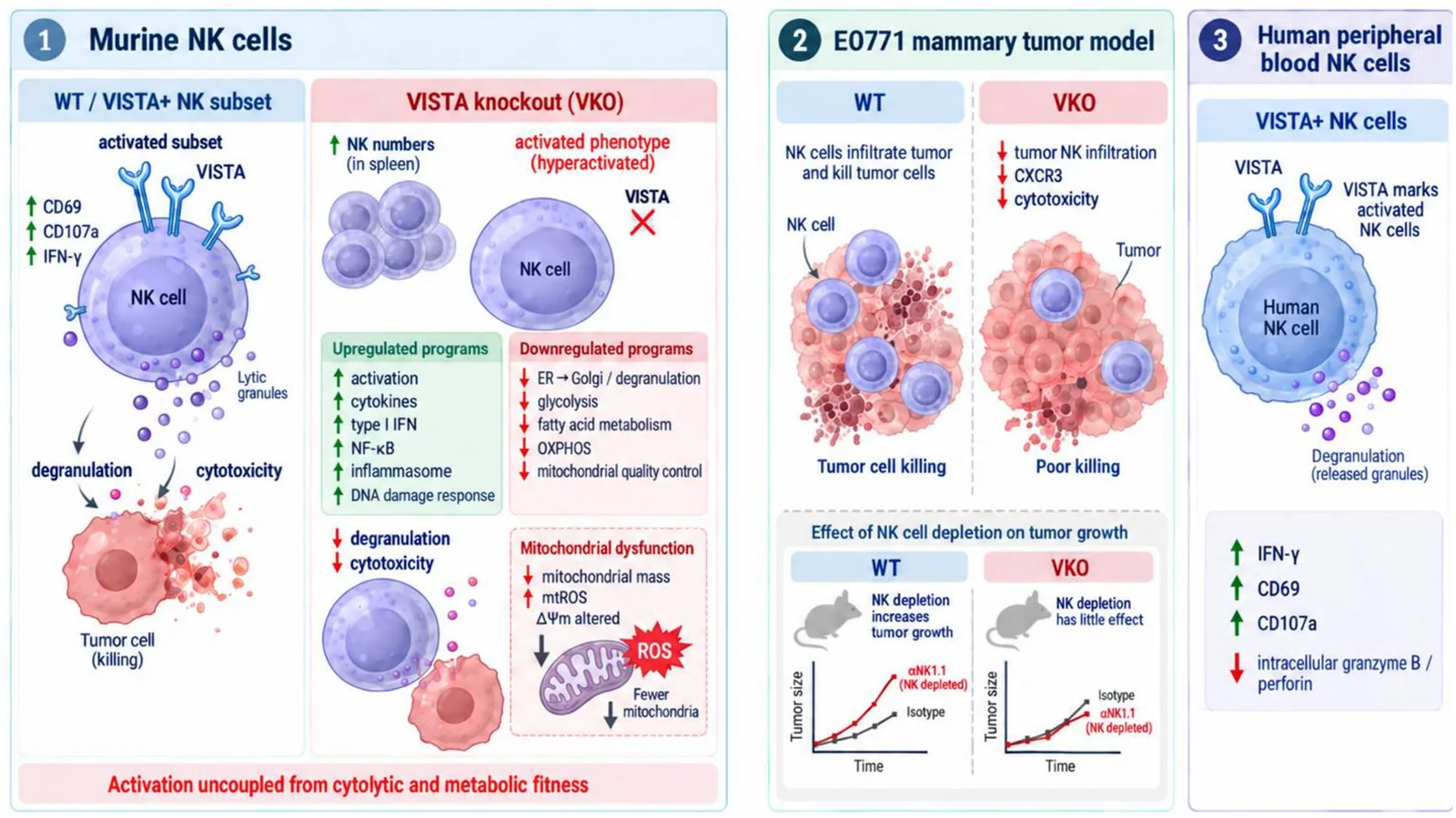

## Introduction

Natural killer (NK) cells are key effectors of innate immunity that mediate host defense through direct cytotoxicity, primarily via perforin and granzyme B (GzmB)-mediated mechanisms, and through the production of inflammatory cytokines, including interferon γ (IFN-γ) and tumor necrosis factor α (TNF-α).^1^^,^^2^ NK cell activity is governed by the integrated balance of activating and inhibitory receptor signals, enabling rapid responses to infected, transformed, or stressed cells while maintaining self-tolerance.^2^^,^^3^ However, in chronic conditions such as viral infections and cancer, sustained activating receptor engagement can drive NK cells into a functionally impaired state in which activation-associated phenotypes become uncoupled from cytotoxic function.^4–7^ In parallel, upregulation of inhibitory receptors under chronic inflammatory conditions may promote an exhausted or dysfunctional NK cell phenotype.^8^^,^^9^

NK cell function is increasingly recognized to be tightly coupled to cellular metabolic requirements.^10^^,^^11^ Under homeostatic conditions, NK cells rely predominantly on oxidative phosphorylation (OXPHOS) to sustain basal activity, whereas activation requires coordinated upregulation of glycolysis and mitochondrial metabolism to meet increased energetic and biosynthetic demands.^12^ These metabolic requirements become particularly critical within the metabolically hostile tumor microenvironment (TME), where nutrient deprivation and oxidative stress impose significant constraints on immune cell effector functions.^13^ Consistent with this concept, accumulating evidence indicates that NK cell persistence and anti-tumor activity depend not only on receptor-mediated activation, but also on the capacity to sustain metabolic adaptability under cellular stress.^7^^,^^14^ Accordingly, defective bioenergetic adaptation may represent a key mechanism by which NK cells acquire activation-associated features without fully preserving cytotoxic function.

These observations further raise the possibility that regulatory pathways governing NK cell activation may also influence the maintenance of metabolic and functional fitness under stress. Although immune checkpoint pathways have been studied predominantly in myeloid cells and T cells, recent studies have demonstrated that NK cells are also subject to checkpoint regulation.^9^^,^^14^^,^^15^ In particular, inhibitory receptors such as LAG-3 and Galectin-9 (Gal-9) have been implicated in shaping NK cell function in a context-dependent manner, including viral infection and cancer.^7^^,^^14^^,^^16^ Collectively, these findings suggest that checkpoint pathways in NK cells do not simply mirror those established in T cells, and that the mechanisms by which checkpoint molecules regulate NK cell functional fitness remain incompletely explored.

Among these checkpoint molecules, V-domain Ig suppressor of T cell activation (VISTA) is an immune checkpoint receptor that restrains inflammatory immune responses, although its signaling mechanisms remain incompletely defined.^15^^,^^17^ VISTA is expressed predominantly by myeloid cells, CD71^+^ erythroid cells (CECs), and T cells.^15^^,^^18^^,^^19^ However, emerging evidence suggests that NK cells may also participate in VISTA-regulated immune responses. For example, in studies of VISTA-targeting agents, depletion of NK cells from peripheral blood mononuclear cells abrogated VISTA agonist-induced myeloid activation,^20^ whereas Fc-competent anti-VISTA antibodies enhanced myeloid responses in a CD16-dependent manner.^21^ However, VISTA expression on NK cells and its role in regulating NK cell phenotype and function remain poorly understood. This concept is particularly important in the context of systemic VISTA deficiency, which is associated with spontaneous immune activation, chronic inflammation, and autoimmunity.^22^^,^^23^ Although such inflammatory environments are expected to reshape NK cell properties, whether they enhance NK effector function or instead promote NK cell dysfunction remains unclear.

Given the growing recognition that NK cell activity depends on coordinated receptor signaling and metabolic fitness, we hypothesized that VISTA deficiency may influence NK cell function in ways not fully captured by conventional activation markers. In this work, we investigated how systemic loss of VISTA shapes NK cell phenotype and function under both homeostatic and tumor-bearing conditions. Using VISTA-knockout (VKO) and wild-type (WT) mice, we combined phenotypic and functional analyses with bulk RNA sequencing to define transcriptional programs associated with NK cell effector functions. We further used the EO771 breast tumor model to determine whether these features persist within the TME and to assess the contribution of NK cells to tumor control. Finally, we evaluated the association between VISTA expression and the phenotype and function of peripheral blood NK cells from healthy controls (HCs) and non-small cell lung cancer (NSCLC) patients.

## Materials and methods

**Animal Ethics statement.** The mouse experimental protocols were approved by the Animal Care and Use Committee at the University of Alberta (protocols AUP0001021 and AUP00002737) in accordance with the guidelines outlined in the Guide for the Care and Use of Laboratory Animals established by The Canadian Council on Animal Care. All animal studies were conducted under AAALAC-accredited ethical standards and are reported in accordance with ARRIVE 2.0 guidelines. Housing, handling, and experimental procedures met all requirements of the Canadian Council on Animal Care. The animals were euthanized using carbon dioxide (CO_2_) in accordance with institutional and regulatory guidelines. Isoflurane was used for the induction and maintenance of anesthesia during tumor inoculation.

**Animals.** Wild-type (WT) C57BL/6 mice were purchased from Charles River Laboratories (Saint-Constant, QC, Canada). C57BL/6 VISTA knockout (Vsir^−/−^) mice were initially provided by Dr. Roy A. Fava.^18^ Both male and female mice were used for immunophenotyping. However, only female mice (8–10 wk) were used for tumor studies.

**Tumor model and NK depletion.** EO771 breast cancer cells were originally obtained from the American Type Culture Collection (ATCC, Manassas, Virginia, USA). Cancer cells were cultured under standard conditions in high-glucose Dulbecco’s Modified Eagle Medium (DMEM; Sigma-Aldrich) supplemented with 10% fetal bovine serum (FBS; Sigma-Aldrich) and 1% penicillin–streptomycin (Sigma-Aldrich) at 37 °C in a humidified incubator with 5% CO_2_. Cancer cells were harvested during the logarithmic growth phase, washed with sterile phosphate-buffered saline (PBS), and resuspended in sterile PBS immediately prior to inoculation. Each mouse was inoculated with $5\times 10^{5}$ EO771 cells resuspended in a total volume of 100 μL PBS into the fourth mammary fat pad under isoflurane anesthesia, as previously reported.^24^

For NK cell depletion, tumor-bearing mice received 200 μg of anti-NK1.1 antibody (clone PK136; Bio X Cell, USA) in 100 μL PBS by intraperitoneal injection every 3 d after tumor inoculation. Control tumor-bearing mice received 100 μL PBS on the same schedule. Following tumor cell inoculation, mice were monitored regularly for tumor growth, overall health status, and signs of distress. Mice were euthanized by CO_2_ inhalation approximately 21 d after tumor inoculation, or when tumors reached the endpoint. Tumors and tissues were collected for downstream experiments.

**Human subjects.** Fresh peripheral blood samples were collected in EDTA-coated tubes from 16 treatment-naive cancer patients with NSCLC and from 12 HCs. The study was approved by the University of Alberta Research Ethics Board (Pro00063463) for HCs and Health Research Ethics Board of Alberta protocol # HREBA.CC-25-0088 for cancer patients. Written informed consent was obtained from all participants prior to sample collection in accordance with the Declaration of Helsinki.

**Isolation of murine immune cells.** Spleens, lungs, livers, and bone marrow (BM) were aseptically harvested from adult mice. Single-cell suspensions from spleen, lung, and liver tissues were prepared by mechanical dissociation between sterile frosted glass slides in 5 mL of 1 × red blood cell (RBC) lysis buffer, followed by filtration through a 40-μm cell strainer according to our protocols.^25^ BM cells were flushed from the bones using a 23G needle, and the suspension was filtered through a 40-μm cell strainer. Cells were subsequently resuspended in high-glucose Dulbecco’s Modified Eagle Medium (DMEM; Sigma-Aldrich) supplemented with 10% fetal bovine serum (FBS; Sigma-Aldrich) and 1% penicillin–streptomycin (Sigma-Aldrich). Immune cells from lung and liver tissues were further enriched by density-gradient centrifugation using Ficoll-Paque PREMIUM 1.084 (MilliporeSigma). Following centrifugation, the mononuclear cell layer (buffy coat) was collected, washed, counted, and prepared for downstream analyses. For tumor tissue processing, tumors were aseptically excised and washed twice with Hanks’ Balanced Salt Solution (HBSS; MilliporeSigma). Tumor tissues were finely minced with sterile scalpels and enzymatically digested in HBSS containing DNase I (20 U; MilliporeSigma) and collagenase type IV (2000 U; MilliporeSigma) at 37 °C for 25 min in a shaking incubator, as previously reported.^24^ Following digestion, cell suspensions were washed with RPMI 1640 medium and sequentially filtered through 70-μm and 40-μm nylon cell strainers to remove residual tissue fragments and aggregates. Tumor-infiltrating mononuclear cells were subsequently isolated by Ficoll-Paque PREMIUM 1.084. The resulting cell fraction was collected, washed, counted, and used for subsequent experiments.

**Isolation of human peripheral blood mononuclear cells (PBMCs).** Human PBMCs were processed within 4 h of collection by Ficoll–Paque Premium 1.077. Briefly, whole blood was diluted with an equal volume of phosphate-buffered saline (PBS), layered over Ficoll–Paque Premium, and centrifuged at 2,000 rpm for 20 min at room temperature with the brake off according to our protocols.^26^^,^^27^ The PBMC layer was carefully collected, washed twice with PBS, counted, and resuspended in complete RPMI-1640 medium (MilliporeSigma) for subsequent experiments.

**Flow cytometry.** Antibodies specific for mouse cell surface antigens and cytokines were purchased mainly from BD Biosciences and Thermo Fisher Scientific. The following antibodies were used for mouse samples: anti-CD3 (145-2C11), anti-CD49b (HMα2), anti-NK1.1 (PK136), anti-VISTA (MIH64), anti-IFN-γ (XMG1.2), anti-Perforin (S16009B), anti-GzmB (NGZB), anti-TNF-α (MP6-XT22), anti-CD107a (1D4B), anti-CD25 (PC61), anti-CD69 (H1.2F3), anti-CD45 (30-F11), anti-CD11b (M1/70), anti-CD27 (LG.3A10). The following antibodies were used for human samples: anti-CD3 (HIT3a), anti-CD16 (B73.1), anti-CD56 (B159), anti-VISTA (IgG2B), anti-IFN-γ (B27), anti-GzmB (GB11), anti-perforin (δG9), anti-CD69 (FN50), ant-CXCR3 (CXCR3-173), and anti-CD107 (H4A3). Dead cells were excluded using the LIVE/DEAD Fixable Aqua Dead Cell Stain Kit (Thermo Fisher Scientific). Cytolytic molecules, cytokines, and the degranulation marker CD107a were measured *ex vivo* at baseline in unstimulated mouse and human NK cells without the use of a Golgi transport inhibitor in total splenocytes or PBMCs, respectively. Thus, the intracellular IFN-γ and TNF-α measurements reflect basal cytokine expression rather than stimulation-induced cytokine production, while perforin and GzmB represent their basal intracellular abundance. CD107a expression was also measured under unstimulated conditions and was not assessed during a target cell–induced degranulation assay. Similarly, Phospho-TBK1 (ser172) (Cell Signaling, cat. #5483) staining was performed on freshly isolated splenocytes from WT and VKO mice, followed by staining with a secondary antibody (Alexa Fluor 647) (abcam, cat. #554714) on NK cells from WT and VKO mice.

Stained cells were fixed and permeabilized using the Cytofix/Cytoperm kit (BD Biosciences), followed by intracellular staining with the appropriate antibodies according to our established protocols.^28^^,^^29^ MitoTracker Green FM Dye (Thermo Fisher Scientific), MitoSOX (Thermo Fisher Scientific), MitoStatus TMRE (BD Pharmingen), and Reactive oxygen species (ROS) (Sigma) staining were performed according to our protocols.^28^^,^^30^ Stained cells were fixed in 4% paraformaldehyde. Data were acquired on an LSRFortessa Cell Analyzer (BD Biosciences) and analyzed using FlowJo version 10.8 software.

**NK cell cytotoxicity assay.** MHC class I-deficient target cells (RMA-S cells; kindly provided by Dr. Kevin Kane, University of Alberta) were labeled with CellTrace™ Violet or CellTrace™ Far Red dye (Thermo Fisher Scientific, USA) according to the manufacturer’s instructions and our protocols.^16^ Labeled target cells were then co-cultured with NK cells isolated from WT or VKO mice at effector-to-target ratios of 5:1, 2:1, 1:1, and 0.5:1. For each condition, $1\;\times \;10^{6}$ WT or VKO NK cells were used to ensure direct comparability between groups. After 4 h of incubation, cells were stained with 7-AAD (Thermo Fisher Scientific) to identify dead target cells, and NK-cell-mediated target cell killing was assessed by flow cytometry.

 Specific killing was calculated as:


$$\text{Specific}\;\text{Killing}\;(\%)=\frac{\text{Dead}\;{\text{CTV}}^{+}\;\text{or}\;{\text{CTR}}^{+}\;\text{target}\;\text{cells}}{\text{Total}\;{\text{CTV}}^{+}\;\text{or}\;{\text{CTR}}^{+}\;\text{target}\;\text{cells}}\times 100\%$$

**Single-Cell RNA Sequencing (scRNA-seq) analysis.** Publicly available scRNA-seq data (GSE119562)^31^ were downloaded from the NCBI GEO database and analyzed in R (v4.5.2) using Seurat (v5.5.0). Cells expressing fewer than 200 genes, more than 4,000 genes, or > 10% mitochondrial transcripts were excluded. Data were normalized, integrated across samples using the Seurat anchor-based integration workflow, and subjected to principal component analysis (PCA), as previously reported.^24^ Unsupervised clustering and UMAP visualization were performed using the first 15 principal components. NK-cell subsets were annotated based on canonical marker gene expression. VISTA^+^ and VISTA^-^ cells were defined by detectable Vsir expression (>0) and used for downstream analyses and visualization.

**Bulk RNA-seq analysis.** NK cells were isolated from the spleens of WT and VKO mice (8 wk old) using the EasySep™ Mouse NK Cell Isolation Kit (STEMCELL Technologies, Vancouver, BC, Canada). Enriched NK cells were further purified by fluorescence-activated cell sorting (FACS) based on the CD3^-^CD49b^+^NK1.1^+^ gating strategy to achieve > 90% purity (Fig. S1A, B). Two WT and four VKO NK-cell biological replicates were collected across independent experimental batches. Total RNA was extracted using the Direct-zol RNA Microprep Kit (Zymo Research), quality assessed by NanoDrop ND-1000, and submitted for bulk RNA sequencing. Libraries were sequenced on an Illumina HiSeq 2500 platform with 150-bp reads. Sequence reads were mapped to GRCm38 using Kallisto pseudo-alignment and differential gene expression analysis was performed in R using the limma-voom pipeline. *P* values were adjusted using the Benjamini–Hochberg method, with adjusted *P* < 0.05 considered significant. Gene Ontology (GO) and Gene Set Enrichment Analysis (GSEA) were performed using clusterProfiler with GO Biological Process gene sets. All samples had an RNA integrity number (RIN) greater than 8, apart from one wild-type sample, which was excluded from analysis due to low RIN.

**Figure 1:**
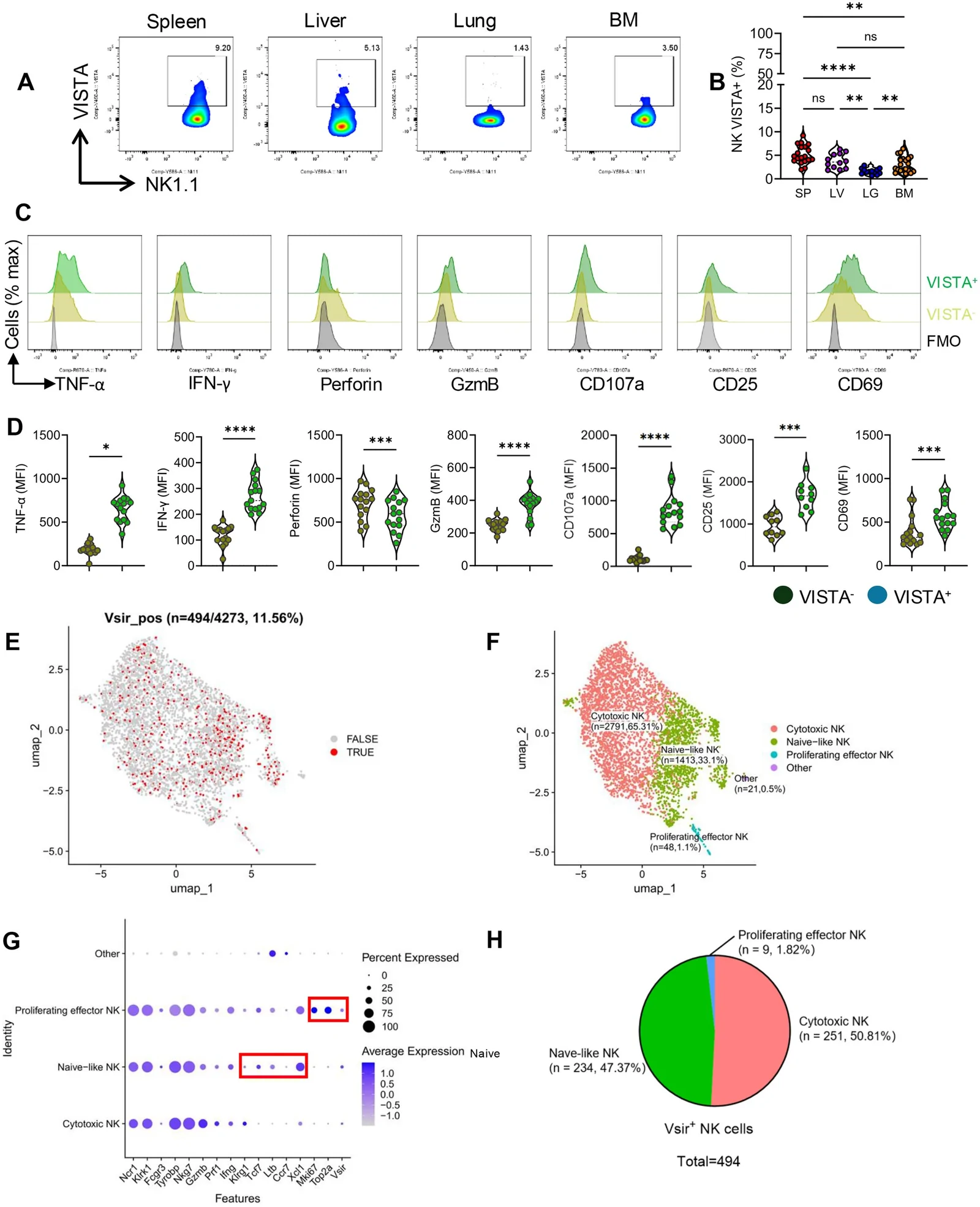
VISTA^+^ NK cells display an activated phenotype. (A) Representative flow cytometry plots, and (B) cumulative data showing the frequency of VISTA^+^ NK cells in the spleen (SP), liver (LV), lung (LG), and bone marrow (BM) of wild type (WT) mice under homeostatic condition. (C) Representative histogram plots, and (D) cumulative data showing the mean fluorescence intensity (MFI) of shown markers in splenic VISTA^−^ versus VISTA^+^ NK cells from WT mice. NK cell effector functions were assessed *ex vivo* under basal, unstimulated conditions in total splenocytes. (E) Feature plot showing the expression of *Vsir* (encoding VISTA) in splenic NK cells pooled from three WT mice. (F) UMAP visualization of splenic NK cell subclusters identified by scRNA seq. (G) Bubble plot showing representative feature genes defining each NK cell subcluster. (H) This plot showing the proportion of *Vsir*+ cells within each NK cell subcluster. Each dot represents a mouse. *P* values were calculated using one-way ANOVA followed by Tukey’s multiple comparison test (B) and Wilcoxon matched pairs (D), * *P* < 0.05, ** < 0.01, *** < 0.001, **** < 0.0001. ns, No significant.

### Statistical analysis

Statistical analyses were performed using GraphPad Prism software (version 9). Data distribution was evaluated using the Shapiro–Wilk normality test. For normally distributed data, one-way or two-way ANOVA was used, as appropriate, followed by (Tukey’s/Dunnett’s) multiple-comparisons test. The Mann–Whitney *U* test was used for comparisons between 2o independent groups when the data did not meet the assumptions of parametric testing, whereas the Wilcoxon matched-pairs signed-rank test was used for paired observations. Correlations between continuous variables were assessed using Pearson correlation analysis, regardless of group classification. Data are presented as mean ± standard error of the mean (SEM). A *P* value < 0.05 was considered statistically significant.

## Results

### VISTA^+^ NK cells exhibit an activated phenotype

To characterize VISTA expression in murine NK cells under physiological conditions, we examined the presence of VISTA across multiple tissues. VISTA was expressed on a subset of NK cells in all tissues examined, with the highest expression observed in the spleen and liver (Fig. 1A, B and Fig. S1A). Given the inhibitory role of VISTA in T cell effector functions,^19^ we anticipated that VISTA expression would be associated with reduced NK cell activity. Unexpectedly, VISTA^+^ NK cells displayed an activated phenotype, with increased expression of cytokines, cytolytic molecules, with the except of perforin, and activation markers compared with their negative counterparts (Fig. 1C, D). Consistent with these observations, analysis of publicly available murine splenic scRNA-seq datasets revealed sparse *Vsir* expression across NK cells, with expression detected in only 11.56% of total NK cells (Fig. 1E). Cytotoxic NK cells constituted the predominant subset, accounting for 65.31% of splenic NK cells (Fig. 1F, G). Notably, the expression pattern of *Vsir* did not distinguish cytotoxic from naïve-like NK cells (Fig. 1H). We also evaluated the effects of sex on the frequency and functionality of VISTA^+^ NK cells but observed no significant differences. These findings support VISTA expression in a subset of NK cells exhibiting an activated phenotype.

### VISTA-deficient NK cells exhibit reduced degranulation capacity and cytotoxicity

To evaluate NK cell effector functions, we compared NK cells from VKO and WT mice. Consistent with a previous report,^19^ VKO mice exhibited splenomegaly compared with WT controls (Fig. 2A, B). Although the frequency of splenic NK cells was reduced in VKO mice (Fig. 2C, D), the increased splenic cellularity resulted in a significantly higher absolute number of NK cells in the spleen (Fig. 2E). Accordingly, NK cell isolation from four pooled spleens per genotype yielded a greater number of NK cells from VKO mice than from WT mice (Fig. 2F). Despite the increased abundance of NK cells in VKO mice, no significant differences in NK cell maturation status were observed between VKO and WT mice, as assessed by the distribution of maturation subsets (Fig. 2G, H).

**Figure 2:**
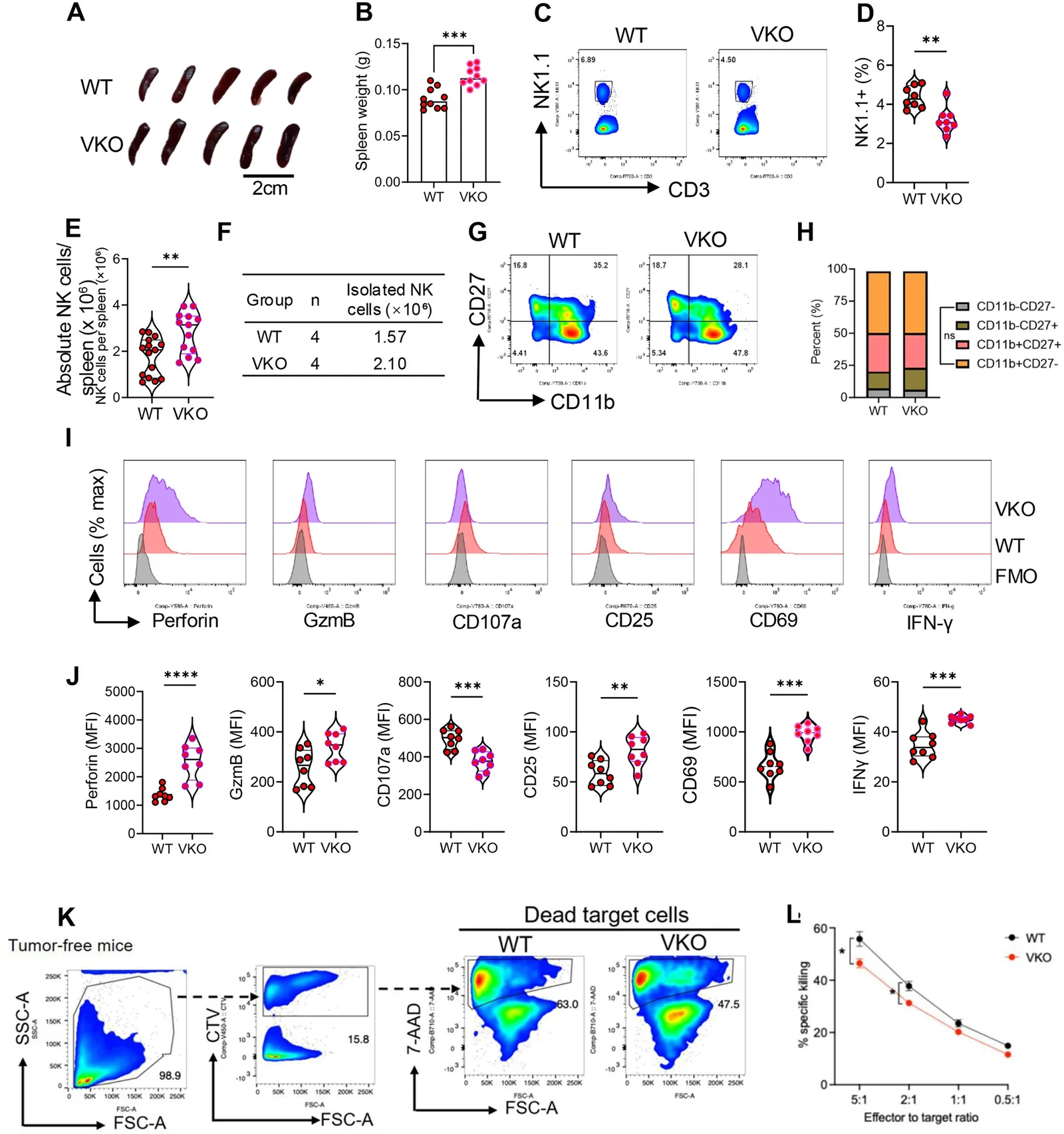
NK cells from VISTA-knockout (VKO) mice exhibit reduced degranulation capacity and cytotoxicity. (A) Representative spleen images, and (B) cumulative quantification of spleen weight in wild-type (WT) and VKO mice. (C) Representative flow cytometry plots, and (D) cumulative data showing the frequency of splenic NK cells in WT and VKO mice. (E) Cumulative data of absolute numbers of splenic NK cells in WT and VKO mice. (F) Summary table showing total number of NK cells isolated from 4 WT and 4 VKO mice. (G) Representative flow cytometry plots, and (H) cumulative quantification of NK cell subsets in WT and VKO mice. (I) Representative flow cytometry histograms, and (J) cumulative data showing mean fluoresce intensity (MFI) of various markers measured in splenic NK cells from VKO versus WT mice. NK cell effector functions were assessed *ex vivo* under basal, unstimulated conditions in total splenocytes. (K) Representative flow cytometry plots showing RMA-S cell viability after coculture with splenic NK cells isolated from WT or VKO mice. (L) Cumulative line plot showing RMA-S cell viability across different effector-to-target ratios (*n* = 3). NK cells were used as effector cells, and RMA-S cells were used as target cells (CTV: CellTrace Violet). Each dot represents a mouse. *P* values were calculated using 2-tailed Mann–Whitney *U* test (B, D, E, J, L) * *P* < 0.05, ** < 0.01, *** < 0.001, **** < 0.0001. ns, No significant.

To further characterize the functional status of VKO NK cells, we compared their cytotoxic and activation profiles with those of WT NK cells. VKO NK cells exhibited increased perforin and granzyme B (GzmB) levels, but significantly reduced CD107a expression (Fig. 2I, J), suggesting that the accumulation of cytotoxic effector molecules is uncoupled from degranulation. In addition, NK cells from VKO mice displayed elevated CD25 and CD69 expression and expressed significantly higher levels of IFN-γ than WT NK cells (Fig. 2I, J). Together, these findings suggest that VISTA deficiency promotes NK cell activation while impairing efficient cytotoxic granule release.

Despite displaying enhanced phenotypic features of activation, purified VKO NK cells exhibited impaired cytotoxicity against RMA-S target cells compared with their WT counterparts *in vitro* (Fig. 2K, L). Collectively, these data demonstrate a functional dissociation in VKO NK cells, whereby increased activation-marker expression and elevated intracellular cytolytic granule content do not translate into efficient degranulation or target-cell killing.

### VISTA deficiency impairs NK cell degranulation and may rewire metabolic transcriptional programs

We next performed bulk RNA-seq to define the transcriptional programs associated with the impaired cytotoxic function of NK cells from VKO mice. Pathway analysis revealed enrichment of gene signatures related to proliferation, differentiation, activation, and cytokine production in VKO NK cells (Fig. 3A), consistent with an activated cellular state. However, this activation program was accompanied by selective negative regulation of NK cell-mediated cytotoxicity (Fig. 3A). Pathways related to endoplasmic reticulum (ER)-to-Golgi membrane transport and degranulation were suppressed, whereas several programs related to intermediate vesicle trafficking and cytoskeletal remodeling were increased (Fig. 3B). Leading-edge gene analysis further supported this model. Representative genes involved in early granule biogenesis and ER-to-Golgi trafficking, including lysosomal-associated membrane protein 1 (*Lamp1*) and neurobeachin (*Nbea*), were downregulated in VKO NK cells (Fig. 3C, D). Similarly, the chemokine receptor, CXCR3, which is important in NK cell trafficking^32^ was notably lower in VKO mice (Fig. 3D). In contrast, genes associated with intermediate vesicle transport and cytoskeletal remodeling, such as *Rab14, Hook, Exoc1,* and *Wasl,*^33^^,^^34^ were upregulated (Fig. 3C, D). At the terminal stage of cytotoxic granule release, synaptotagmin-3 (*Syt3*) was reduced, but *Gzmb* and its endogenous inhibitor *Serpinb9* (also known as proteinase inhibitor 9 or SPI-6 in mice)^35^ were increased (Fig. 3C, D). Together, these transcriptional changes suggest a dysregulated cytotoxic secretory pathway in VKO NK cells, in which altered vesicle trafficking and cytoskeletal remodeling may fail to restore efficient degranulation.

**Figure 3:**
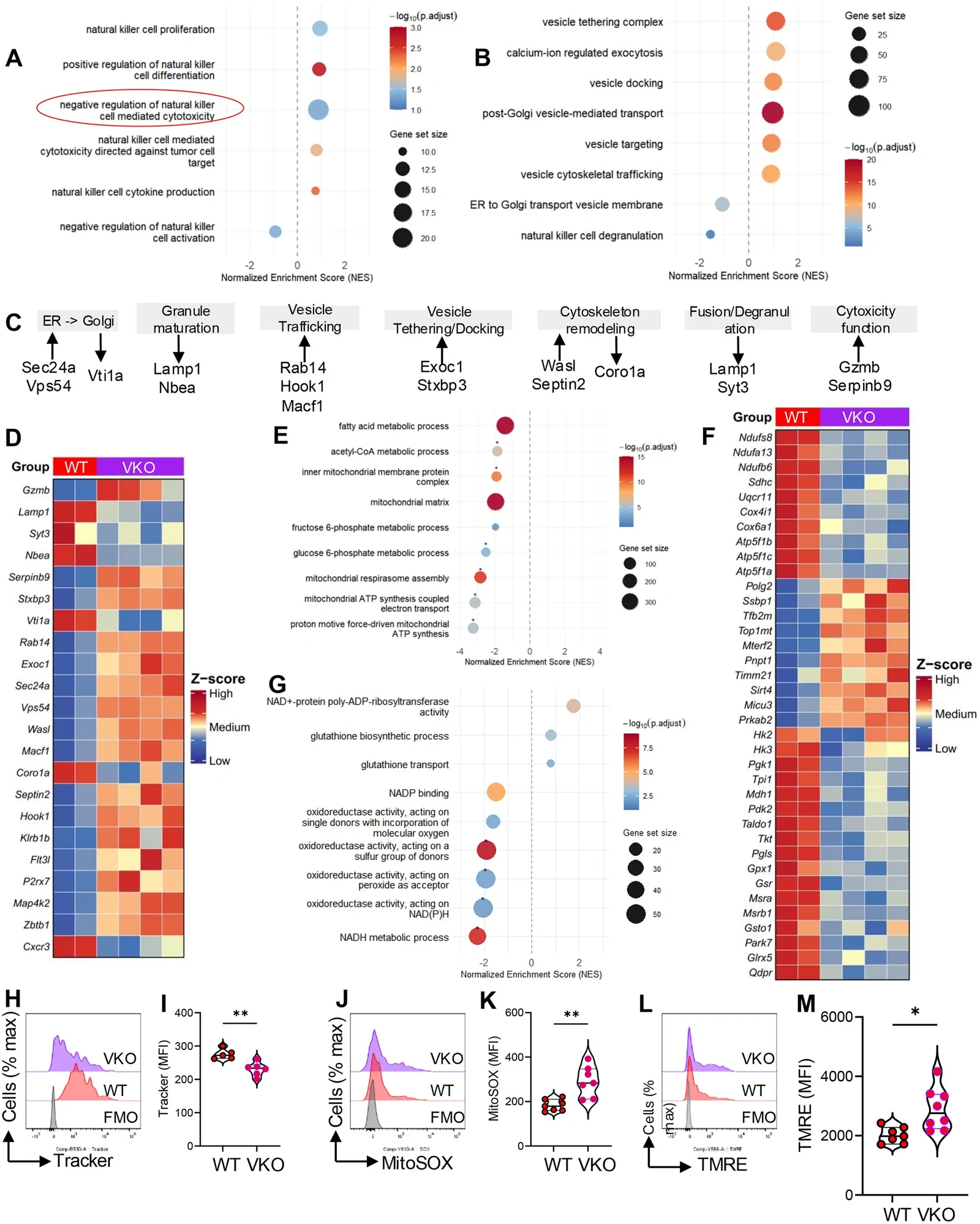
Transcriptional alterations in VISTA-knockout (VKO) NK cells reveal impaired degranulation-associated programs and mitochondrial dysfunction. (A) Bubble plot showing GSEA-GO enrichment of NK cell function related pathways in VKO NK cells. Positive normalized enrichment scores (NES > 0) indicate pathways enriched in VKO NK cells, whereas negative normalized enrichment scores (NES < 0) indicate pathways enriched in WT NK cells relative to VKO NK cells. Bubble color indicates pathway significance, and bubble size represents the number of genes included in each pathway. (B) Bubble plot showing enrichment of pathways associated with NK cell exocytosis. (C) Schematic illustration and (D) heatmap showing significantly altered feature genes involved in the exocytosis process. (E) Bubble plot showing enrichment of pathways related to glycolysis and mitochondrial function. Asterisks indicate pathway enrichment significance. (F) Bubble plot showing enrichment of redox-associated pathways involved in reactive oxygen species (ROS) homeostasis. (G) Heatmap showing leading-edge genes associated with mitochondrial function, glycolysis, and redox regulation. (H) Representative flow cytometry histograms, and (I) cumulative quantification of mitochondrial mass in splenic NK cells from WT and VKO mice, measured by MitoTracker Green. (J) Representative flow cytometry histograms, and (K) cumulative quantification of mitochondrial ROS levels in splenic NK cells from WT and VKO mice, measured by MitoSOX. (L) Representative flow cytometry histograms, and (M) cumulative quantification of mitochondrial membrane potential in splenic NK cells from WT and VKO mice, measured by tetramethylrhodamine ethyl ester (TMRE). Each dot represents a mouse. *P* values were calculated using 2-tailed, Mann–Whitney *U* test (I, K, M) * *P* < 0.05, **.

Given the dependence of NK cell effector function on metabolic fitness, we next investigated whether VISTA deficiency reshapes NK cell metabolism. Our bulk RNA-seq revealed broad repression of metabolic programs in VKO NK cells compared with WT controls, including pathways involved in glycolysis, fatty acid metabolism, and mitochondrial bioenergetics. In particular, genes associated with fatty acid metabolism, mitochondrial assembly, and glycolysis processes including fructose-6-phosphate, glucose-6-phosphate, and acetyl-CoA metabolism, were markedly downregulated (Fig. 3E). This was accompanied by coordinated suppression of key glycolytic genes, including *Hk2, Hk3, Pgk1,* and *Tpi1*^36^^,^^37^ (Fig. 3F). Mitochondrial ATP-generating pathways were also substantially reduced, including proton-motive-force-driven ATP synthesis, electron-transport-coupled oxidative phosphorylation, and respirasome assembly (Fig. 3E). Consistent with this, transcripts encoding electron transport chain complexes I-V were broadly decreased in VKO NK cells (Fig. 3F). In addition, VKO NK cells exhibited dysregulated mitochondrial quality-control pathways, characterized by increased *Fundc1* expression but reduced expression of genes associated with the PINK1 pathway, including *Pink1, Park7, Ubb, Ubc,* and *Iscu*^38–40^ (Fig. S2A). These findings suggest that VISTA deficiency may promote a metabolically constrained NK cell state characterized by reduced glycolytic capacity, impaired mitochondrial bioenergetics, and altered mitochondrial stress adaptation.

In parallel, bulk RNA-seq analysis revealed broad disruption of redox homeostasis in VKO NK cells. Although glutathione biosynthesis and transport programs were upregulated, suggesting a compensatory response to oxidative stress, pathways associated with NADH/NADPH-dependent oxidoreductase activity, peroxide detoxification, and sulfur-redox repair pathways were suppressed (Fig. 3G). Consistent with these findings, the expression of genes involved in glucose-derived NADPH generation, including *Pgls, Tkt,* and *Taldo1*, was reduced, along with that of key redox-buffering genes such as *Gpx1, Gsr, Park7,* and *Glrx5* (Fig. 3F). These data suggest that VISTA deficiency may induce an incomplete redox-adaptive state in NK cells, whereby enhanced glutathione-related programs are insufficient to restore antioxidant capacity, potentially contributing to persistent mitochondrial stress.

To functionally validate the transcriptional alterations observed in VKO NK cells, we assessed mitochondrial content and function in steady-state splenic NK cells from WT and VKO mice. VKO NK cells exhibited reduced mitochondrial mass, as determined by MitoTracker staining (Fig. 3H, I), along with increased mitochondrial ROS production measured by MitoSOX staining (Fig. 3J, K). Despite the reduction in mitochondrial content, VKO NK cells displayed elevated mitochondrial membrane potential (Fig. 3L, M), consistent with mitochondrial hyperpolarization.^36^ Collectively, these findings indicate that VISTA deficiency induces mitochondrial dysfunction in NK cells, characterized by reduced mitochondrial mass, increased ROS accumulation, and altered mitochondrial polarization.

### VISTA deficiency induces replication stress-associated cytosolic nucleic acid sensing and inflammatory remodeling in NK cells

Mitochondrial stress can propagate to broader cellular stress programs;^38^^,^^41^ therefore, we next assessed biosynthetic and DNA-damage responses in VKO NK cells. Gene ontology analysis revealed enrichment of replication-linked damage pathways, including those involved in DNA replication regulation, helicase activity, and double-strand break processing (Fig. 4A). Consistent with these findings, VKO NK cells showed increased expression of DNA-damage and replication stress-associated genes, including *Atm, Atr, Chek1, Brca2,* and *Blm*, together with chromatin-regulatory factors *Kdm5a, Kdm6a, Cbx5,* and *Smarcad1*^42–44^ (Fig. 4B). In contrast, translation-related pathways, including tRNA aminoacylation and SSU/LSU rRNA maturation, were suppressed (Fig. 4A), accompanied by reduced expression of *Rps19, Rps14, Rpl3, Lars2,* and *Fars2*^45^^,^^46^ (Fig. 4B). These data suggest a shift away from global protein synthesis toward a stress-surveillance state characterized by replication stress-associated transcriptional programs and impaired translational capacity.

**Figure 4:**
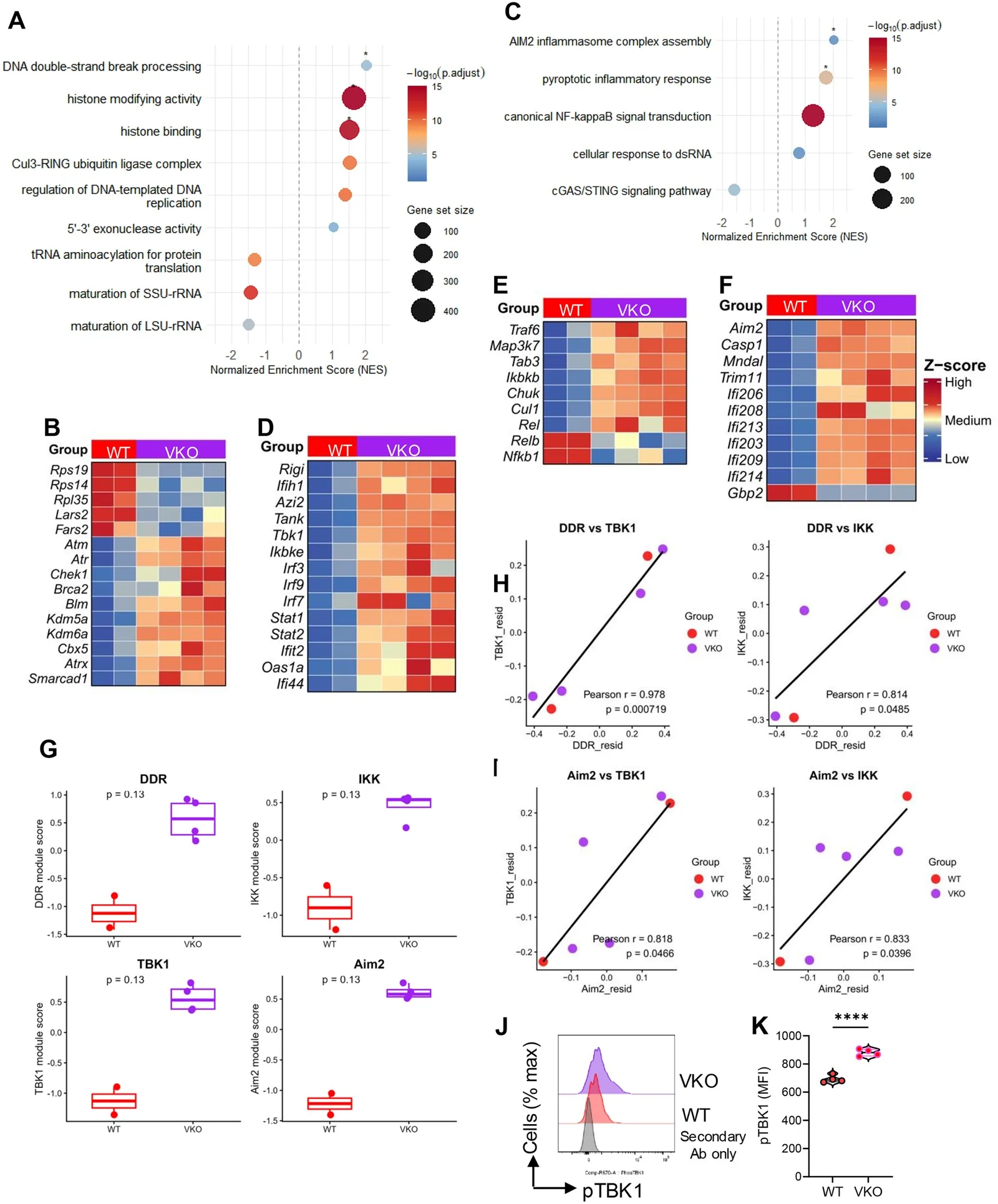
Transcriptional alterations in VISTA-knockout (VKO) NK cells reveal replication stress-associated and innate inflammatory programs. (A) Bubble plot showing enrichment of pathways related to genomic instability, including DNA damage response, DNA replication, and translational machinery–associated pathways. (B) Heatmaps showing significantly altered genes associated with translational machinery and DNA damage related pathways. (C) Bubble plot showing enrichment of pathways associated with downstream responses to genomic stress, including cellular responses to dsRNA, AIM2 inflammasome signaling, pyroptotic inflammatory responses, and NF-ĸB signaling pathways. (D) Heatmap showing significantly altered core genes within the TBK1 signaling module, (E) within the IKK signaling module, and (F) within the AIM2 inflammasome module. (G) Comparison of DDR, IKK, TBK1, and AIM2 module scores between wild type (WT) and VKO NK cells. (H) Correlation analysis between DDR and TBK1 or IKK module scores following adjustment for group effects. (I) Correlation analysis between AIM2 and TBK1 or IKK module scores following adjustment for group effects. (J) Representative histogram plots, and (K) cumulative data showing Phospo-TBK1 in WT and VKO mice. Each dot represents a mouse. *P* values were calculated using 2-tailed, Mann–Whitney *U* test (G, K) and Pearson correlation (H, I).

Given that genotoxic stress can promote the release or exposure of nucleic acids capable of activating innate immune sensors,^47^ we next examined whether these inflammatory programs were altered in VKO NK cells. Consistent with elevated DNA-damage signatures, VKO NK cells were enriched for nucleic-acid-sensing and inflammatory pathways, including AIM2 inflammasome complex assembly, responses to double-stranded RNA, canonical NF-ĸB signaling, and pyroptosis-associated inflammation (Fig. 4C).

VKO NK cells showed coordinated activation of two major innate inflammatory axes. First, the TBK1-type I interferon (IFN) module was increased, as reflected by elevated expression of RLR sensors (*Rigi* and *Ifih1*), TBK1-associated adaptors (*Azi2* and *Tank*), core kinases (*Tbk1* and *Ikbke*), interferon-regulatory factors (*Irf3, Irf7, Irf9, Stat1,* and *Stat2*), and interferon-stimulated genes (*Ifit2, Oas1a,* and *Ifi44*) (Fig. 4D).

In parallel, the IKK-NF-ĸB pathway was enhanced, marked by increased expression of upstream signaling components, including *Traf6, Map3k7/Tak1*, and Tab3, together with the core IKK complex subunits *Ikbkb* and Chuk (Fig. 4E). This was accompanied by remodeling of NF-ĸB subunit expression, characterized by increased *Rel* but reduced *Relb* and *Nfkb1* genes (Fig. 4E). In addition, VKO NK cells upregulated *Aim2*, PYHIN family members (*Mndal, Ifi203, Ifi209, Ifi214*), and *Casp1* (Fig. 4F), supporting activation of cytosolic DNA-sensing and inflammasome-associated programs.^48^^,^^49^

We next calculated module scores for DDR, IKK-NF-ĸB, TBK1-type I interferon, and AIM2-associated genes. VKO NK cells showed a trend toward higher module scores than WT NK cells across these inflammation-related programs (Fig. 4G). After adjustment for genotype-associated effects, DDR scores remained significantly and positively correlated with both TBK1-IFN and IKK-NF-ĸB scores, with the strongest association observed along the DDR-TBK1-IFN axis (Fig. 4H). In contrast, DDR scores were not significantly associated with AIM2 scores (Fig. S2B). AIM2 scores were instead positively correlated with both TBK1-IFN and IKK-NF-ĸB modules (Fig. 4I). To confirm that the observed upregulation of TBK1- and type I IFN-associated transcripts translated into functional pathway activation, we evaluated the phosphorylation status of key signaling enzyme in NK cells via flow cytometry. In alignment with our transcriptomic data, NK cells at the baseline from VKO mice displayed elevated levels of phosphorylated TBK1 (p-TBK1, Ser172) (Fig. 4J, K). These findings validate that the TBK1 kinase module is enzymatically active and functionally driving downstream type I IFN signaling at the protein level. Together, these data suggest that VISTA deficiency may link DNA damage-associated stress to innate inflammatory remodeling in NK cells, with a strong connection to the TBK1-type I interferon pathway.

### VISTA deficiency uncouples NK cell accumulation and activation from cytotoxic competence during EO771 tumor challenge

Next, we investigated the role of VKO NK cells using the EO771 mammary tumor model. Tumor-bearing VKO mice exhibited splenomegaly relative to WT mice (Fig. 5A, B), consistent with their steady-state phenotype (Fig. 2A). Although the frequency of splenic NK cells was lower (Fig. 5C, D), the absolute number of NK cells per spleen was significantly higher due to the enlarged spleen size in VKO mice (Fig. 5E), consistent with what we observed in tumor-free mice (Fig. 2D, E). Phenotypic profiling of splenic NK cells from tumor-bearing mice showed that VKO NK cells expressed higher levels of GzmB, perforin, TNF-α, IFN-γ, and CD69, while surface CD107a was significantly lower than in WT mice (Fig. 5F, G). These data indicate that VKO NK cells remain activated in the tumor-bearing mice but exhibit impaired degranulation capacity. In parallel, VKO NK cells showed reduced mitochondrial mass (Fig. 5H, I), increased mitochondrial ROS production (Fig. 5J, K), and impaired mitochondrial depolarization capacity (Fig. 5L, M), consistent with mitochondrial dysfunction.^38^ Functionally, splenic NK cells from tumor-bearing VKO mice exhibited reduced cytotoxicity against target cells compared with WT NK cells (Fig. 5N, O), confirming impaired cytotoxic capacity. Together, these findings suggest that VISTA deficiency sustains an activated but cytotoxicity-impaired NK cell state during tumor challenge.

**Figure 5:**
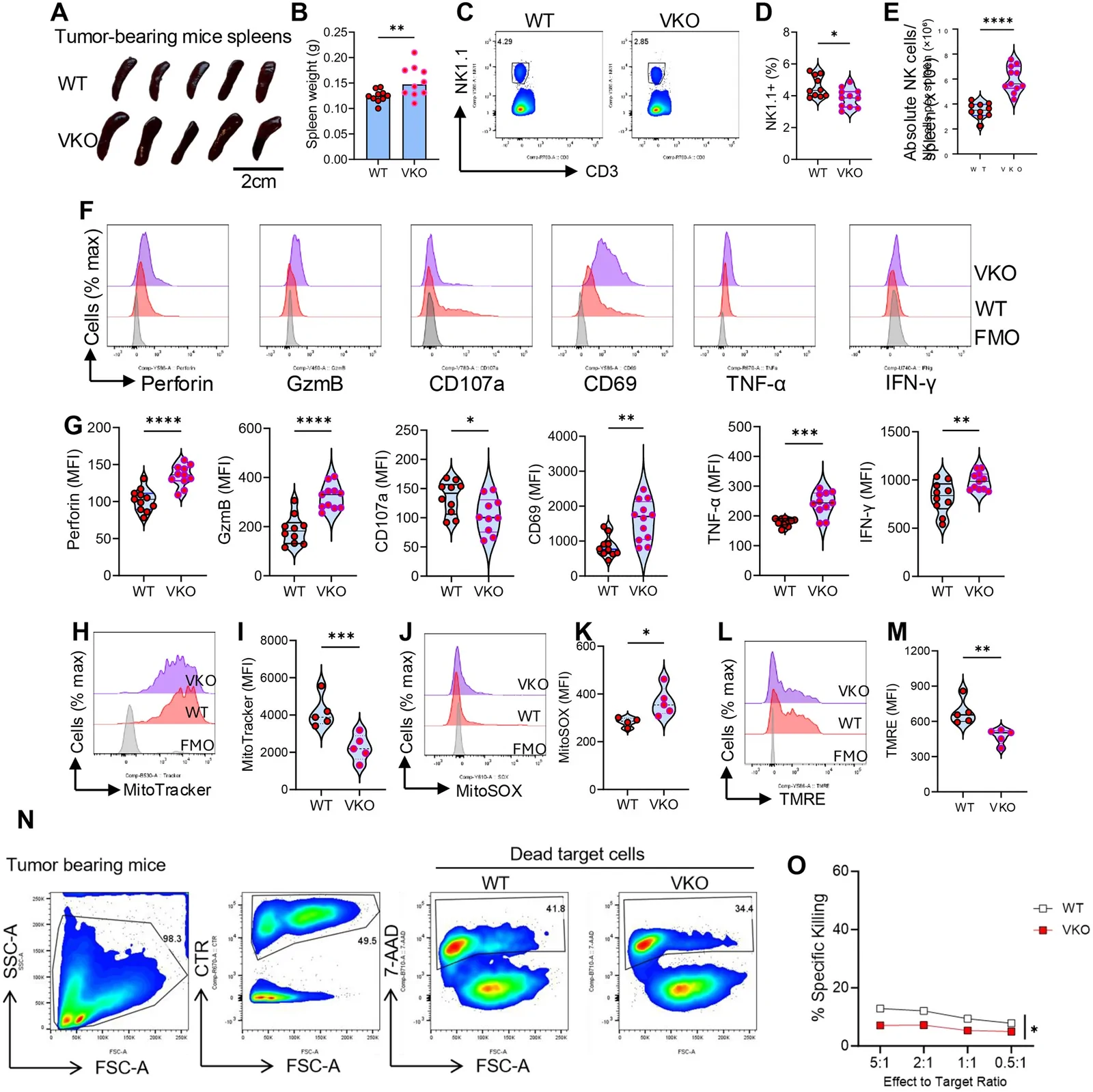
Splenic VISTA-knockout (VKO) NK cells retain an activated phenotype but exhibit impaired cytotoxic function in tumor-bearing mice. (A) Representative spleen images, and (B) cumulative data of spleen weights from WT and VKO tumor-bearing mice. (C) Representative flow cytometry, and (D) cumulative data showing the frequency, and (E) absolute numbers of splenic NK cells in WT and VKO tumor-bearing mice. (F) Representative flow cytometry histograms, and (G) cumulative quantification of indicated markers in splenic NK cells from tumor-bearing WT and VKO mice. (H) Representative flow cytometry histograms, and (I) cumulative quantification of mitochondrial mass in splenic NK cells from tumor-bearing WT and VKO mice. (J) Representative flow cytometry histograms, and (K) cumulative quantification of mitochondrial reactive oxygen species (ROS) levels in splenic NK cells from tumor-bearing WT and VKO mice. (L) Representative flow cytometry histograms, and (M) cumulative quantification of mitochondrial membrane potential in splenic NK cells from tumor-bearing WT and VKO mice. (N) Representative flow cytometry plots, and (O) cumulative line plot showing RMA-S cell viability across different effector-to-target ratios following coculture with splenic NK cells isolated from tumor-bearing WT or VKO mice (*n* = 3). Each dot represents a mouse. *P* values were calculated using 2-tailed, Mann–Whitney *U* test (B, D, E, G, I, K, M) and 2-way ANOVA (O) * *P* < 0.05, ** < 0.01, *** < 0.001, **** < 0.0001. MFI, Mean fluorescence intensity.

### NK cells may not be the primary contributors to tumor control in VKO mice

The inhibitory role of VISTA in suppressing CD8^+^ T cell effector functions, as evidenced by the robust antitumor immune response observed in VKO mice, has been well established;^50^^,^^51^ Consistent with these reports, VKO mice developed smaller EO771 tumors than WT controls (Fig. 6A, B). Under these conditions, VKO tumors contained a significantly lower frequency of infiltrating NK cells compared with WT tumors (Fig. 6C, D). However, VKO tumor-infiltrating NK cells expressed higher levels of TNF-α, IFN-γ, perforin, and GzmB, together with increased CD25 but reduced CD69 and CD107a expression (Fig. 6E, F). These findings indicate that NK cells in the VKO TME adopt an activated phenotype with impaired degranulation capacity.

**Figure 6:**
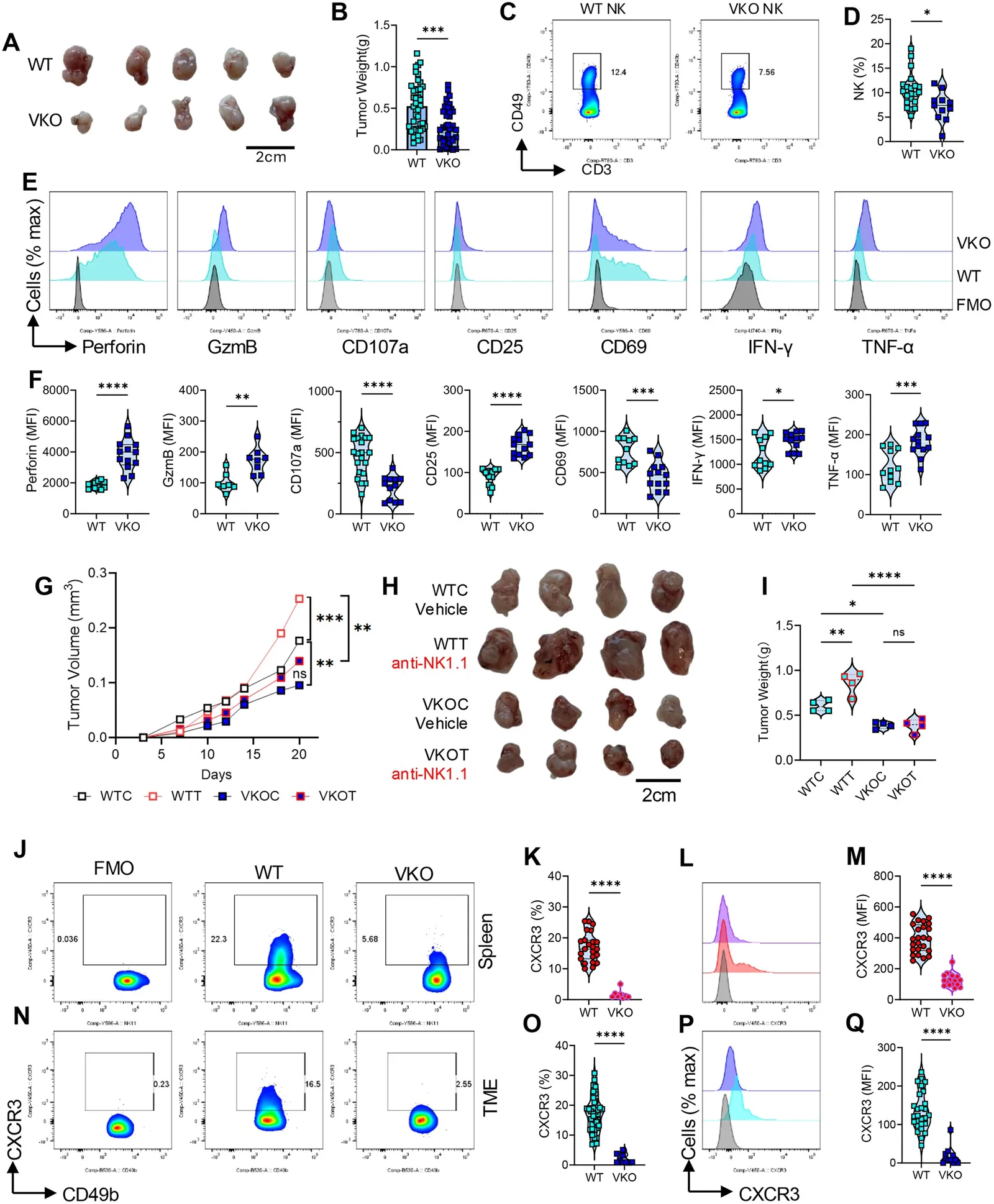
Tumor regression is mediated by NK cells from WT mice but not by NK cells from VISTA-knockout (VKO) mice. (A) Representative tumor images, and (B) cumulative quantification of tumor weights in WT and VKO mice at the endpoint. (C) Representative flow cytometry plots, and (D) cumulative quantification of NK cell frequencies within the TME of WT and VKO mice. (E) Representative flow cytometry histograms, and (F) cumulative quantification of indicated markers in tumor-infiltrating NK cells from WT and VKO mice. (G) Cumulative tumor growth curves of EO771 tumor-bearing WT or VKO mice treated with anti-NK1.1 antibody or vehicle control. (H) Representative tumor images from WT and VKO mice treated with vehicle or anti-NK1.1 antibody at the experimental endpoint. (I) Cumulative quantification of tumor volume and tumor weight at the experimental endpoint in WT and VKO mice treated with vehicle or anti-NK1.1 antibody. This is a representative data from two independent experiments. (J) Representative flow cytometry plots, and (K) cumulative data of the frequency of CXCR3 expressing splenic NK cells from WT and VKO mice. (L) Representative histograms, and (M) cumulative data showing the intensity of CXCR3 expression in splenic NK cells from WT and VKO mice. (N) Representative flow cytometry plots, and (O) cumulative data of the frequency of CXCR3 expressing NK cells within the TME from WT and VKO mice. (P) Representative histograms, and (Q) cumulative data showing the intensity of CXCR3 expression in NK cells within the TME of WT and VKO mice. Each dot represents a mouse. *P* values were calculated using 2-tailed, Mann–Whitney *U* test (B, D, F, K, M, O, Q) and 1-way ANOVA (I), * *P* < 0.05, ** < 0.01, *** < 0.001, **** < 0.0001. ns, No significant; MFI, Mean fluorescence intensity. FMO, Fluorescence minus one; WTC, Wild type control; WTT, WT-treated; VKOC, VKO control; and VKOT, VKO-treated.

To determine whether NK cells contribute to tumor control in VKO mice, we depleted NK cells in EO771 tumor-bearing VKO mice and control mice using anti-NK1.1 neutralizing antibody (Fig. S2C). We found that this treatment resulted in a significant reduction in the frequency of intratumoral NK1.1^+^CD49b^+^ NK cells (Fig. S3A, B), whereas the overall frequencies of CD4^+^ and CD8^+^ T cells remained largely unchanged (Fig. S3C). Interestingly, we found that although depletion of NK cells had no effect on tumor progression in VKO mice, it resulted in a significant increase in tumor size in WT mice (Fig. 6G-I).

Additionally, we observed that while NK cell depletion had minimal effects on the frequency of tumor-infiltrating T cells expressing co-inhibitory receptors (e.g., PD-1, TIM-3, and TIGIT) in WT mice, it reduced the frequencies of PD-1^+^, TIM-3^+^, and TIGIT^+^ CD4^+^ T cells, but not CD8^+^ T cells, in VKO mice (Fig. S3D, E).

### Reduced CXCR3 expression in VKO NK cells may impair-NK cell infiltration into the TME

Given the significantly higher absolute number of splenic NK cells in VKO mice but their markedly lower abundance within the TME, we sought to determine the underlying mechanism. NK cell trafficking and tissue homing are regulated by multiple chemokine receptors; however, CXCR3 has been reported to play a key role in promoting NK cell infiltration into TME.^32^^,^^52^ Therefore, we evaluated CXCR3 expression on NK cells from WT and VKO mice. Interestingly, under homeostatic condition, splenic NK cells from VKO mice exhibited significantly lower frequency of CXCR3^+^ cells and reduced CXCR3 expression compared with those from WT mice (Fig. S4A–D). A similar reduction in both the frequency (Fig. 6J, K) and expression intensity of CXCR3 (Fig. 6L, M) was observed in splenic NK cells from tumor-bearing VKO mice relative to WT controls. Furthermore, NK cells isolated from the TME of VKO mice displayed significantly lower CXCR3 frequency (Fig. 6N, O). and expression intensity (Fig. 6P, Q) than their WT counterparts. Collectively, these findings suggest that reduced CXCR3 expression may impair NK-cell trafficking and infiltration into the TME, thereby contributing to the decreased accumulation of NK cells in tumors of VKO mice. However,

### VISTA expression identifies activated NK cell subsets in humans

Finally, we investigated the frequency of and VISTA expression on different NK cell subsets in the PBMCs of HCs and NSCLC patients. Notably, no differences in the proportions of NK cell subsets were observed between HCs and cancer patients (Fig. 7A–C). Although CD16^-^CD56^hi^ and CD16^hi^CD56^+^ NK cells expressed negligible amounts of VISTA, we found that CD16^hi^CD56^-^, CD16^lo^CD56^−^, and CD16^−^CD56^lo^ NK cell subsets expressed elevated levels of VISTA in both HCs and cancer patients (Fig. 7D, E).

**Figure 7:**
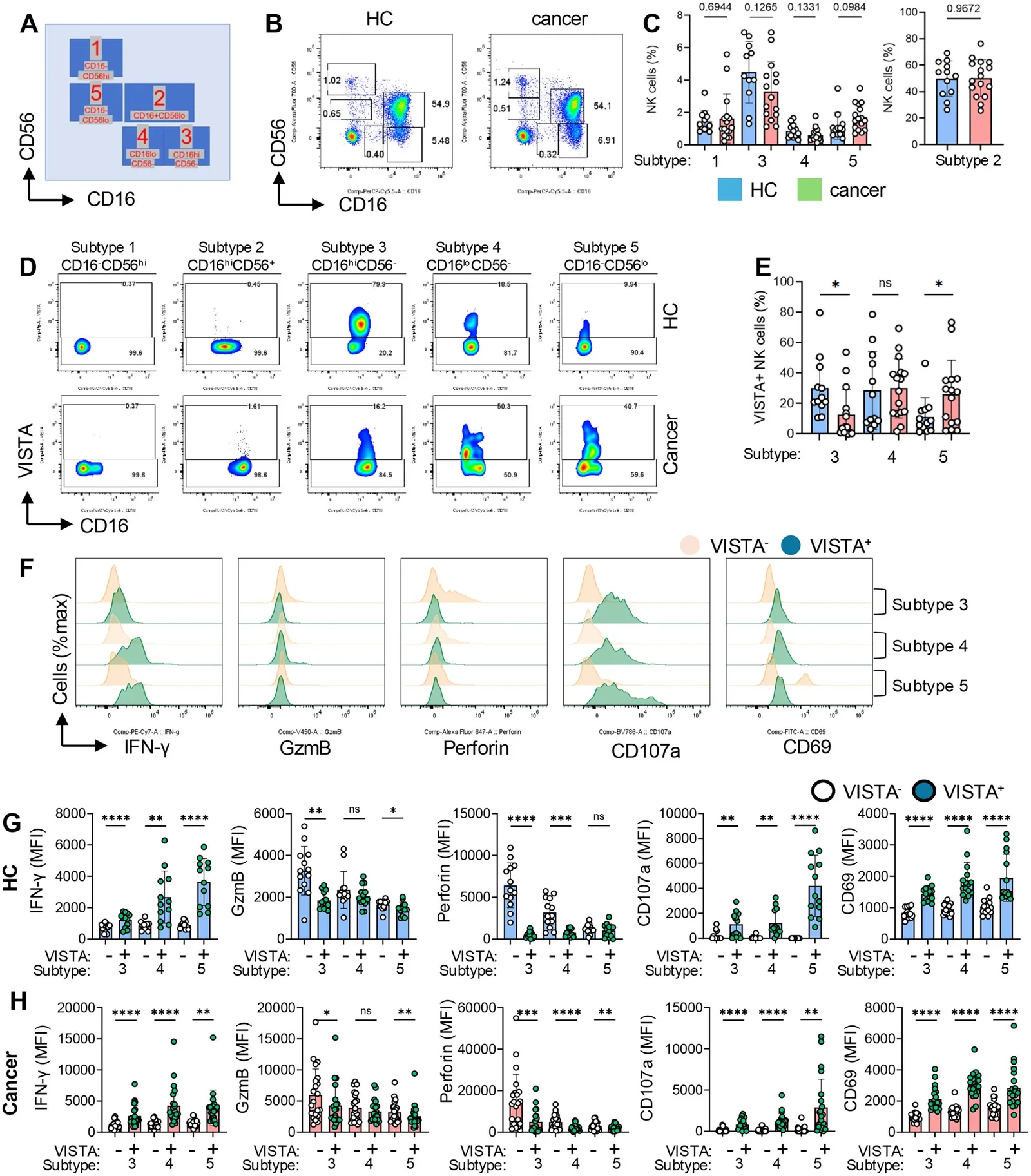
VISTA^+^ NK cells in human subjects exhibit an activated phenotype. (A) Schematic plot of NK cell subsets. (B) Representative flow cytometry plots, and (C) cumulative data of the proportion of NK cell subsets in human healthy controls (HCs) versus non-small cell lung cancer (NSCLC) patients. (D) Representative plots, and (E) cumulative data of the frequency of VISTA^+^ NK cell subsets in HCs and cancer patients. (F) Representative histograms, and (G) cumulative data of indicated activation markers in VISTA^+^ versus VISTA^-^ NK cells in HCs, and (H) cancer patients. Each dot represents a human study subject. *P* values were calculated using 2-tailed, Mann–Whitney *U* test (C, E) and Wilcoxon matched-pairs (G, H) * *P* < 0.05, ** < 0.01, *** < 0.001, **** < 0.0001. ns, No significant; MFI, Mean fluorescence intensity.

Interestingly, VISTA^+^ NK cell subsets exhibited an activated phenotype characterized by higher expression of IFN-γ, CD69, and CD107a but lower GzmB and perforin in both HCs and cancer patients (Fig. 7F–H). The lower intracellular GzmB and perforin levels in VISTA^+^ NK cells may reflect enhanced degranulation and prior release of cytolytic granules. However, the lower intracellular levels of perforin and GzmB observed in VISTA-positive NK cells cannot be attributed exclusively to previous degranulation and may also reflect altered synthesis or replenishment of cytolytic proteins, differences in NK-cell maturation, or variations in activation state. Further functional studies are required to distinguish among these possibilities.

## Discussion

In this study, we identify a paradoxical NK cell state in VKO mice, characterized by phenotypic hyperactivation coexisting with an impairment of cytolytic capacity. This phenotype was observed not only in splenic NK cells under homeostatic conditions but also in tumor-bearing mice. Similar dissociations between surface activation markers and actual cytotoxic function have previously been reported in defined NK cell subsets expressing LAG-3 and PD-1.^7^^,^^13^ Our findings extend this conceptual paradigm to VISTA, showing that the genetic loss of this unique immune checkpoint drives the emergence of an activated but cytolytically constrained NK cell subset.

While the inhibitory role of VISTA in T cell effector functions is well established,^53^ systemic VISTA deficiency triggers widespread immune activation, characterized by marked splenomegaly, spontaneous lymphoproliferation, and chronic autoinflammatory conditions.^19^^,^^54^ Therefore, the chronic systemic inflammation in VKO hosts may expose the NK cell compartment to persistent, low-level stimulation, thereby reshaping NK cell phenotype, metabolism, and effector potential. Under these conditions, the expression of classical surface activation markers, such as CD25, CD69, and intracellular cytokines (e.g., IFN-γ and TNF-α) does not indicate functional fitness but rather highlights a chronic inflammatory state.^7^ In this context, NK cells exposed to hypoxia or ex vivo expanded NK cells provide useful conceptual comparators, as both represent metabolically and functionally reprogrammed NK cell states.^5^ VKO NK cells shared several features with hypoxic NK cells, including an activated phenotype associated with reduced cytotoxicity and elevated intracellular ROS levels.^5^ However, in contrast to hypoxic or ex vivo-expanded NK cells, VKO NK cells exhibited concurrent reductions in both glycolytic activity and mitochondrial oxidative phosphorylation. These findings suggest that chronic inflammatory stimulation associated with VISTA deficiency drives a functionally constrained NK cell state, possibly through impaired glycolytic and mitochondrial metabolic fitness.^55^

Mechanistically, this functionally constrained state was associated with impaired degranulation capacity. Effective NK cell degranulation requires the coordinated execution of multiple sequential processes, including lytic granule biogenesis and maturation, ER-to-Golgi transport, vesicle trafficking, granule tethering and docking at the immunological synapse, cytoskeletal remodeling, and ultimately SNARE-mediated membrane fusion and degranulation.^55^ Consistent with this requirement, suppression of ER-to-Golgi transport and degranulation-related programs in VKO NK cells suggests disruption of the cytotoxic secretory machinery, thereby compromising effective NK cell cytotoxicity. For example, the downregulation of *Nbea* in VKO NK cells suggests a failure in the structural retention and sorting of cytotoxic proteins within lytic organelles, potentially leading to disordered trafficking.^56^

In parallel, activation of nucleic acid-sensing pathways, together with inflammasome-related and type I interferon transcriptional programs, suggests that endogenous stress-induced innate inflammatory signaling^57^ may contribute to the activated phenotype observed in VKO NK cells.

Additionally, in NK cells, lytic granules are specialized lysosome-related organelles (LROs) that must fuse with the plasma membrane to release granzymes and perforin.^58^ The selective repression of *Syt3* in VKO NK cells represents a loss of a critical calcium sensor required for lytic vesicle exocytosis at the immunological synapse and may contribute to their failure to upregulate CD107a during target cell exposure.

Moreover, our bulk RNA-seq demonstrated that VKO NK cells concurrently upregulated *Gzmb* and its specific nucleocytoplasmic serine protease inhibitor, *Serpinb9.*^35^ The upregulation of *Serpinb9* in VKO NK cells is consistent with a protective, homeostatic feedback loop triggered by high intracellular GzmB reserves.

NK cell cytolytic exocytosis and cytokine production are energetically demanding processes that require the rapid upregulation of glycolysis and OXPHOS to fuel vesicle trafficking, cytoskeletal remodeling, and target cell lysis.^59^ In VKO NK cells, we observed a broad, coordinated transcriptional repression of core metabolic programs, including the downregulation of primary glycolytic genes (*Hk2*, *Hk3*, *Pgk1*, *Tpi1*) and electron transport chain (ETC) complexes I–V.

This bioenergetic shutdown was accompanied by structural and functional mitochondrial dysfunction. Steady-state VKO NK cells exhibited a significant loss of mitochondrial mass, marked accumulation of mitochondrial ROS, and persistent mitochondrial hyperpolarization.

AMP-activated protein kinase (AMPK), a central sensor of cellular energy status, may represent an additional mechanistic link between chronic VISTA deficiency and the metabolic dysfunction observed in VKO NK cells. AMPK coordinates cellular responses to energy stress by promoting metabolic pathways that restore ATP availability and support mitochondrial homeostasis.^60^ Thus, dysregulated AMPK signaling could potentially contribute to the concurrent suppression of glycolytic and oxidative metabolism and, consequently, to the impaired cytolytic function of VKO NK cells. Although AMPK activity was not directly assessed in the present study, this pathway warrants investigation as a potential mediator of the immunometabolic consequences of VISTA deficiency.

This phenotype matches the metabolic and mitochondrial quality-control defects documented in autoimmune diseases, particularly systemic lupus erythematosus (SLE).^61^

Under normal physiological conditions, mitochondria generate low levels of ROS to modulate cellular signaling pathways. However, in VKO NK cells, the downregulations of mitophagy-associated quality-control pathways (repressing Pink1, Park7, and Iscu) may prevent the clearance of damaged organelles.^62^ The subsequent failure to sustain antioxidant defense programs (characterized by the downregulation of glucose-derived NADPH-generating genes *Pgls*, *Tkt*, and *Taldo1*, alongside *Gpx1* and *Gsr*) may leave VKO NK cells under continuous oxidative stress.

Faced with persistent mitochondrial ROS and a lack of metabolic resources, VKO NK cells exhibited severe replication stress and genomic instability, marked by a robust DNA damage response (DDR) signature.^63^ VKO NK cells upregulated key double-strand break repair and replication checkpoint genes, including *Atm*, *Atr*, *Chek1*, *Brca2*, and *Blm*. To survive this genotoxic crisis, VKO NK cells suppressed global translation-related pathways while engaging in a stress-surveillance state.

Importantly, our transcriptomic and correlation analyses revealed that this chronic genotoxic stress may lead to the activation of cytosolic nucleic acid-sensing and innate inflammatory pathways in VKO NK cells. DNA double-strand breaks induce the transcription of interferon-stimulated genes (ISGs) and type I interferons, a process that requires ATM and NF-kappaB activation.^64^ In VKO NK cells, this connection is supported by the strong, positive correlation between DDR scores and both the TBK1-type I interferon (*Rigi*, *Ifih1*, *Tbk1*, *Irf3*, *Irf7*) and IKK-NF-kappaB (*Traf6*, *Ikbkb*, *Chuk*) signaling modules.

Furthermore, VKO NK cells displayed upregulation of the AIM2 inflammasome complex (*Aim2*, *Mndal*, *Ifi203*, *Ifi209*, *Ifi214*, *Casp1*). AIM2-associated DNA sensors detect double-stranded DNA in the cytosol to initiate inflammasome assembly and pyroptosis-associated inflammation.^65^ Our correlation analysis indicated that AIM2 scores do not correlate directly with DDR scores but instead correlate with both TBK1-type I IFN and IKK-NF-kappaB modules. This supports a model of sterile inflammatory remodeling in which DNA damage may drive type I interferon expression, which then initiates a self-amplifying feedback loop that promotes AIM2 inflammasome assembly.^65^^,^^66^

Crucially, while type I interferons have antiviral and antitumor properties, persistent DNA sensing and chronic type I interferon signaling can paradoxically suppress NK cell function and promote functional exhaustion.^67^ Chronic type I IFN signaling and DNA-sensing-induced innate pathways may accelerate cell death programs, driving NK cells into a state of chronic activation without cytolytic competence.^68^ This sterile, self-amplifying inflammatory loop may explain how VISTA deficiency leads to chronic NK cell activation alongside severe metabolic and cytolytic defects. These findings raise the possibility that the impaired ATP-generating capacity of VKO NK cells represents, at least in part, an exhaustion-like metabolic state induced by chronic stimulation. Such a state could limit the metabolic flexibility required to sustain both glycolytic and oxidative ATP production during NK cell effector responses.

To determine whether these tumor-infiltrating NK cells contributed to tumor control in VKO mice, we selectively depleted the NK cell compartment. In WT mice, NK cell depletion accelerated tumor progression, confirming that functional NK cells are required for baseline tumor control. In contrast, NK cell depletion in VKO mice had no effect on tumor growth kinetics, confirming that despite their abundance and activated phenotype, VKO NK cells do not contribute directly to tumor clearance *in vivo*.

In the VKO TME, the accumulation of chronically activated but cytolytically inactive NK cells may exert aberrant, pro-inflammatory bystander effects that promote or sustain exhaustion pathways in tumor-infiltrating helper T cells. This identifies a novel immunoregulatory role for NK cells under chronic checkpoint deficiency, where dysfunctional NK cells can continue to shape the local adaptive immune landscape.

Consistently, human VISTA^+^ NK cells exhibited an activated phenotypic profile, characterized by high expression of IFN-γ, CD69, and CD107a, but lower intracellular pools of perforin and GzmB. This is distinct from the cytolytically blocked murine VKO phenotype, which represents systemic genetic deletion. Instead, this phenotypic profile is consistent with active effector function; human NK cells may have a high baseline degranulation capacity, allowing them to rapidly discharge their granzyme and perforin cargo, leaving intracellular reserves depleted. This conserved relationship indicates that under normal physiological conditions, VISTA acts as a marker of NK cell activation, suggesting a potential role for VISTA in modulating human NK cell homeostasis during immune responses.

Collectively, these findings raise the possibility that VISTA signaling must be maintained within an appropriate physiological range to preserve NK cell metabolic fitness and effector function. Rather than functioning solely as an inhibitory checkpoint, VISTA may contribute to maintaining immune homeostasis by preventing sustained or excessive NK cell activation that can ultimately become metabolically detrimental. Thus, both excessive inhibitory signaling and complete loss of VISTA-mediated regulation may disrupt NK cell function through distinct mechanisms, highlighting the importance of balanced VISTA signaling for optimal NK cell homeostasis.

A primary limitation of this study is that systemic VISTA deficiency makes it difficult to definitively separate NK cell-intrinsic signaling from host-extrinsic effects driven by the chronic inflammatory environment. Under steady-state conditions, *Vsir* transcript expression was detected in only a small fraction (11.56%) of WT splenic NK cells, showing a preferential enrichment in proliferating and naive-like states relative to mature cytotoxic states. This sparse basal transcription suggests that the dysfunctional NK cell phenotype in VKO mice is shaped primarily by host-extrinsic factors, specifically, the chronic inflammatory milieu of the VISTA-deficient host. This interpretation is supported by the widespread genomic, metabolic, and quality-control defects observed in VKO NK cells, which match the chronic immune-metabolic stress seen in systemic autoimmune diseases. Moreover, lower abundance of NK cells in TME indicates a potential defect in NK-cell trafficking in VKO mice. CXCR3 has been recognized as a critical chemokine receptor that mediates NK-cell migration toward CXCL9-, CXCL10-, and CXCL11-rich tumor sites.^52^^,^^69^ Consistent with this role, we observed reduced CXCR3 expression on splenic NK cells under both homeostatic and tumor-bearing conditions, as well as on tumor-infiltrating NK cells from VKO mice. These data raise the possibility that diminished CXCR3 expression may contribute to impaired trafficking of VKO NK cells to tumors, thereby reducing their accumulation within the TME. However, the abundance of intratumoral NK cells is determined not only by recruitment but also by several other processes, including survival, proliferation, apoptosis, retention within the tumor, and local expansion. Because these possibilities were not directly examined in the current study, the reduced NK-cell accumulation cannot be attributed solely to altered CXCR3 expression. Functional migration studies, together with analyses of NK-cell survival, proliferation, apoptosis, and retention within tumors, will be required to define the relative contribution of each mechanism. Thus, our findings identify reduced CXCR3 expression as one potential contributor to, rather than a definitive mechanism responsible for, the decreased NK-cell infiltration observed in VKO tumors.

Another limitation of our study is that VISTA expression was not assessed in PBMCs from breast cancer patients, which would have been more directly relevant to the EO771 mammary tumor model. This was due to the lack of access to breast cancer patient samples; therefore, PBMCs from patients with NSCLC were used as an available human cancer cohort.

In conclusion, our findings demonstrate that systemic VISTA deficiency does not act as a simple immunological accelerator. Instead, the loss of VISTA uncouples NK cell phenotypic activation from its mandatory metabolic and secretory foundations. In the absence of VISTA, chronic inflammatory bystander effects subject NK cells to persistent stimulation, driving mitochondrial decay, redox failure, replication stress, and sterile cytosolic nucleic acid-sensing pathways. This locks NK cells into a state of chronic activation without cytolytic competence, which persists within the TME.

## Supplementary Material

vkag282_Supplementary_Data

## Data Availability

All data related to this study are included in the main and supplemental figures. Additionally, our bulk RNAseq data are publicly available at the NCBI website with the accession number PRJNA1480600 or GSE336028.
