# Supplementary material for: VISTA deficiency drives an activated NK cell phenotype with impaired cytotoxic function
Source: J Immunol. 2026 Nov 13;215(11):vkag282. doi: 10.1093/jimmun/vkag282 (PMC13768109; doi:10.1093/jimmun/vkag282)
Supplement: Supplementary file 1 [file vkag282_supplementary_data.pdf]

S Figure 1

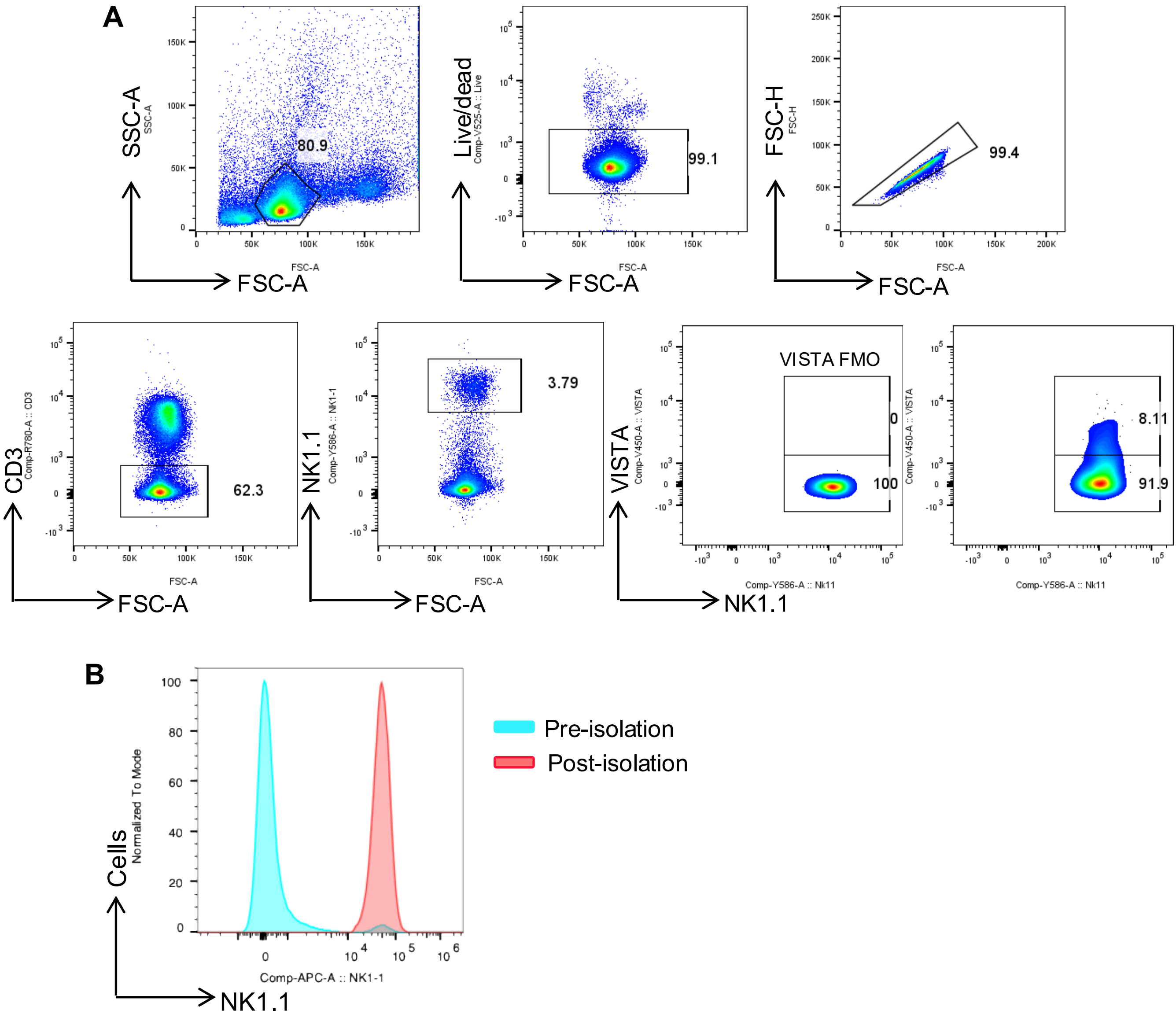

S Fig 1. The gating strategy for the identification of NK cells in mice tissues. (B) Representative plot showing the purity of NK cells pre-and-post isolation for RNA-seq.

S Figure 2

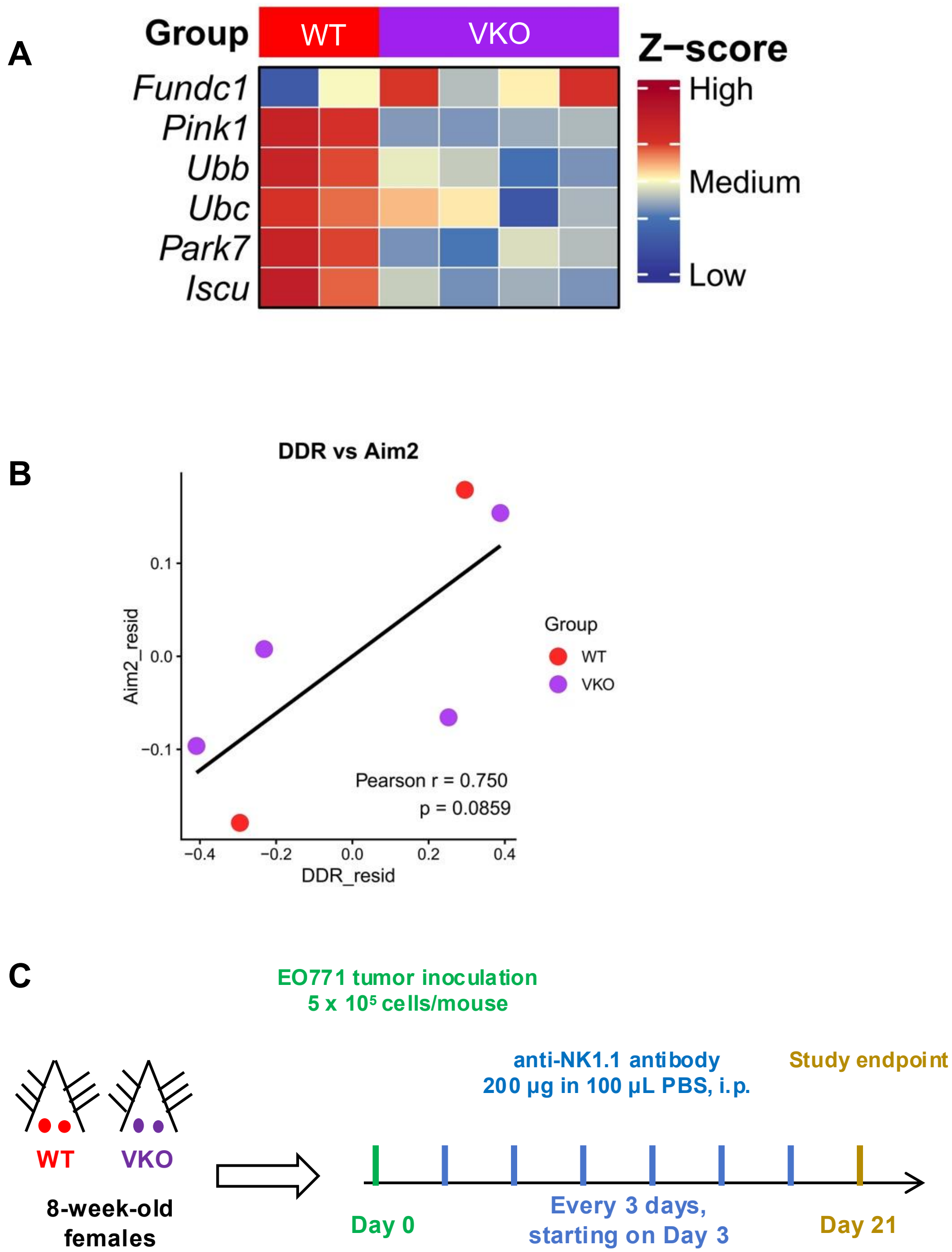

S Fig 2. A) Heatmap showing significantly altered core genes involved in the Pink1-Park7 mitophagy signaling pathway. **B**) Correlation analysis between DDR and AIM2 module scores following adjustment for group effects. **C**) Schematic illustrating the anti-NK1.1 treatment regimen.

S Figure 3

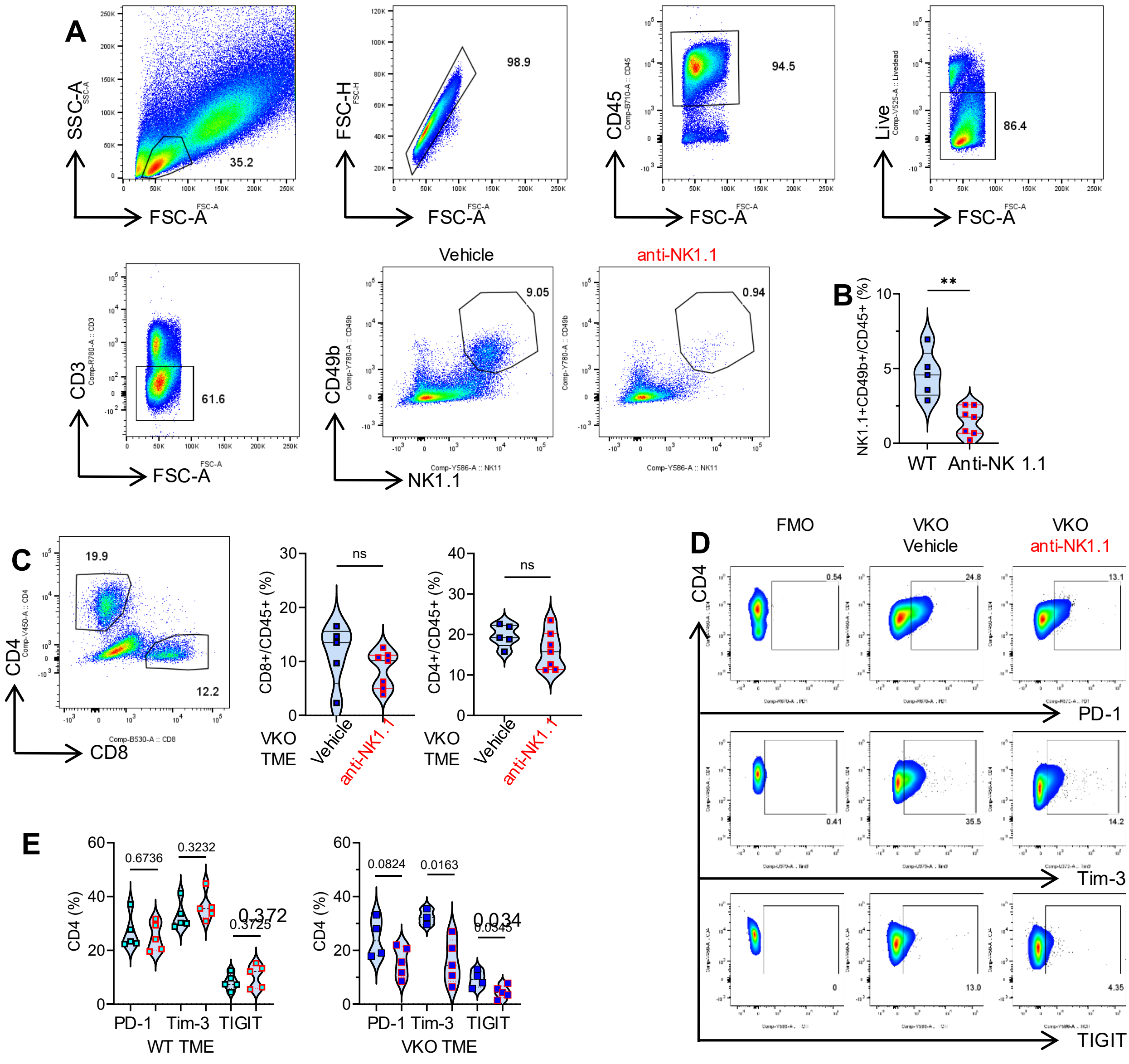

S Fig 3. **A**) Flow cytometry gating strategy, and **B**) cumulative data for tumor-infiltrating NK cells in WT and treated mice. **C**) Representative flow cytometry plots and cumulative quantification of CD4^{+} and CD8^{+} T cells among CD45^{+} immune cells within the VKO TME under vehicle or anti-NK1.1 treatment.

D) Representative flow cytometry plots, and **E**) cumulative quantification of PD-1, Tim-3, and TIGIT expression on CD4 T cells within the TME of VKO mice under vehicle or anti-NK1.1 treatment.

S Figure 4

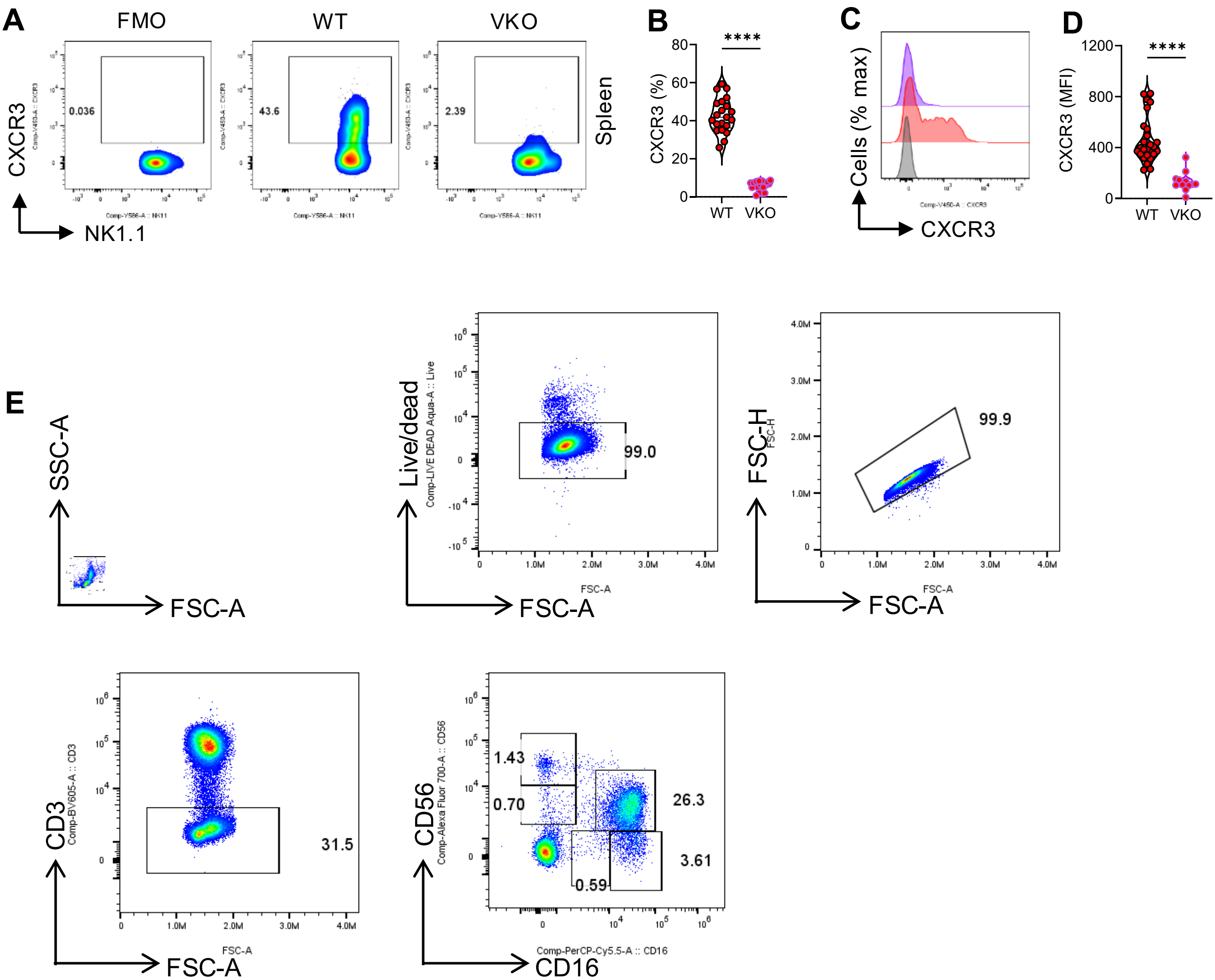

S Fig 4. **A**) Representative flow cytometry plots, and **B**) cumulative data showing the frequency of CXCR3-expressing splenic NK cells in WT and VKO mice homeostatic condition. **C**) Representative histograms, and **D**) cumulative data of the intensity of CXCR3 in splenic NK cells under homeostatic condition. **E**) Flow cytometry gating strategy for human NK cell subsets in PBMCs.
